# Use of IFNγ/IL10 Ratio for Stratification of Hydrocortisone Therapy in Patients With Septic Shock

**DOI:** 10.3389/fimmu.2021.607217

**Published:** 2021-03-09

**Authors:** Rainer König, Amol Kolte, Olaf Ahlers, Marcus Oswald, Veiko Krauss, Daniela Roell, Oliver Sommerfeld, George Dimopoulos, Iraklis Tsangaris, Eleni Antoniadou, Neeraja Jaishankar, Holger Bogatsch, Markus Löffler, Markus Rödel, Marina Garcia-Moreno, Lorena Tuchscherr, Charles L. Sprung, Mervyn Singer, Frank Brunkhorst, Michael Oppert, Herwig Gerlach, Ralf A. Claus, Sina M. Coldewey, Josef Briegel, Evangelos J. Giamarellos-Bourboulis, Didier Keh, Michael Bauer

**Affiliations:** ^1^Integrated Research and Treatment Center, Center for Sepsis Control and Care, Jena University Hospital, Jena, Germany; ^2^Institute of Infectious Diseases and Infection Control, Jena University Hospital, Jena, Germany; ^3^Department of Anesthesiology and Operative Intensive Care Medicine (CCM, CVK), Berlin Institute of Health, Charité - Universitätsmedizin Berlin, Freie Universität Berlin, Humboldt-Universität zu Berlin, Berlin, Germany; ^4^2nd Department of Critical Care Medicine, Medical School, National and Kapodistrian University of Athens, Athens, Greece; ^5^Intensive Care Unit, “George Gennimatas” Thessaloniki General Hospital, Thessaloniki, Greece; ^6^Clinical Trial Centre, Leipzig University, Leipzig, Germany; ^7^Institute for Medical Informatics, Statistics and Epidemiology, Leipzig University, Leipzig, Germany; ^8^Institute of Medical Microbiology, Jena University Hospital, Jena, Germany; ^9^Department of Anesthesiology and Critical Care Medicine, Hadassah Hebrew University Medical Center, Jerusalem, Israel; ^10^Division of Medicine, Bloomsbury Institute of Intensive Care Medicine, University College London, London, United Kingdom; ^11^Department of Emergency and Intensive Care Medicine, Klinikum Ernst von Bergmann, Potsdam, Germany; ^12^Department of Anesthesia, Operative Intensive Care Medicine, and Pain Management, Vivantes Neukölln Hospital, Berlin, Germany; ^13^Department of Anesthesiology and Intensive Care Medicine, Jena University Hospital, Jena, Germany; ^14^Septomics Research Center, Jena University Hospital, Jena, Germany; ^15^Department of Anesthesiology, University Hospital, Ludwig Maximilian University of Munich, Munich, Germany; ^16^4th Department of Internal Medicine, Medical School, National and Kapodistrian University of Athens, Athens, Greece

**Keywords:** sepsis, hydrocortisone, immune therapy, machine learning, theranostics, steroids, IFNgamma/IL10

## Abstract

Large clinical trials testing hydrocortisone therapy in septic shock have produced conflicting results. Subgroups may benefit of hydrocortisone treatment depending on their individual immune response. We performed an exploratory analysis of the database from the international randomized controlled clinical trial Corticosteroid Therapy of Septic Shock (CORTICUS) employing machine learning to a panel of 137 variables collected from the Berlin subcohort comprising 83 patients including demographic and clinical measures, organ failure scores, leukocyte counts and levels of circulating cytokines. The identified theranostic marker was validated against data from a cohort of the Hellenic Sepsis Study Group (HSSG) (*n* = 246), patients enrolled in the clinical trial of Sodium Selenite and Procalcitonin Guided Antimicrobial Therapy in Severe Sepsis (SISPCT, *n* = 118), and another, smaller clinical trial (Crossover study, *n* = 20). In addition, *in vitro* blood culture experiments and *in vivo* experiments in mouse models were performed to assess biological plausibility. A low serum IFNγ/IL10 ratio predicted increased survival in the hydrocortisone group whereas a high ratio predicted better survival in the placebo group. Using this marker for a decision rule, we applied it to three validation sets and observed the same trend. Experimental studies *in vitro* revealed that IFNγ/IL10 was negatively associated with the load of (heat inactivated) pathogens in spiked human blood and in septic mouse models. Accordingly, an *in silico* analysis of published IFNγ and IL10 values in bacteremic and non-bacteremic patients with the Systemic Inflammatory Response Syndrome supported this association between the ratio and pathogen burden. We propose IFNγ/IL10 as a molecular marker supporting the decision to administer hydrocortisone to patients in septic shock. Prospective clinical studies are necessary and standard operating procedures need to be implemented, particularly to define a generic threshold. If confirmed, IFNγ/IL10 may become a suitable theranostic marker for an urging clinical need.

## Introduction

Though prospective, randomized, controlled multicenter trials have consistently reported faster shock resolution (1, 2), the utility of “low-dose” hydrocortisone (HC) in patients with septic shock remains controversial. Whereas, two French studies reported outcome benefit from a combination of hydrocortisone plus oral fludrocortisone (3, 4), the pan-European CORTICUS trial and the 5-country ADRENAL trial found no survival effect from hydrocortisone alone (2, 5). Possible explanations for this disparity included differences in mortality risk in the populations with a two-fold higher risk of mortality in the French control group [of ref. (1)] compared to CORTICUS (61 vs. 31%, respectively), and an increase in superinfections, variations in other aspects of clinical management, and genetic variations. Of note, a subset analysis of the ADRENAL trial indicated survival benefit from hydrocortisone in Australasian patients, no effect in British and Danish patients, and a trend to harm in patients enrolled in Saudi Arabia (2). It is increasingly recognized that patients presenting in septic shock are hyper-inflamed yet at the same time immunosuppressed (6–8). Corticosteroids are traditionally considered to induce immune suppression *via* the glucocorticoid receptor (GR) and its repressive effect on pro-inflammatory transcription factors such as AP-1 and NFκB (9). Thus, patients in an overall state of immunosuppression may be potentially compromised by administration of an immunosuppressive drug. This argument is, however, complicated by increasing evidence implicating corticosteroids and GRs in immune-reconstitutive processes (10, 11).

In human monocytes corticosteroid treatment induced expression of innate immune-related genes, such as Toll-like Receptors and anti-inflammatory genes (10, 11). In macrophages and derived cell lines glucocorticoids induced a central component of the inflammasome (NLRP3) and, upon stimulation with endotoxin (lipopolysaccharide, LPS), induced secretion of pro-inflammatory cytokines such as Tumor Necrosis Factor alpha (TNFα) (11). Furthermore, glucocorticoid-dependent NLRP3 induction resulted in sensitization of innate immune cells to extracellular ATP and, thus, in an enhanced ATP-mediated secretion of pro-inflammatory cytokines following endotoxin stimulation (12). This immune-activating role of corticosteroids has been described as a response to acute stress enhancing the peripheral immune response, whereas chronic corticosteroid exposure leads to immune suppression (13, 14). These diverging effects of corticosteroids support the need for biomarkers to guide their application.

We applied machine learning to physiological and laboratory data from patients enrolled within a sub-population of the CORTICUS study to search for a potential theranostic marker for hydrocortisone treatment. Using the ratio of serum interferon gamma (IFNγ) to interleukin 10 (IL10), we were able to identify specific sub-cohorts with increased and decreased survival upon treatment. Furthermore, we explored the predictive utility of this biomarker in further datasets of septic shock patients and performed experimental studies regarding this marker as a function of pathogen challenge.

## Materials and Methods

### Included Cohorts and Studies

The discovery dataset based on the Berlin subcohort of the CORTICUS trial. The CORTICUS study was approved by local Ethics Committees (No: 153/2001). Written informed consent was obtained from patients, proxies or their legal representatives. Eligible patients were enrolled if they met the following inclusion criteria: clinical evidence of infection, evidence of a systemic response to infection, the onset of shock within the previous 72 h and hypoperfusion or organ dysfunction attributable to sepsis. Notable exclusion criteria included an underlying disease process with a poor prognosis, life expectancy <24 h, long-term immunosuppression and treatment with long-term corticosteroids within the past 6 months or with short-term corticosteroids within the past 4 weeks. Detailed eligibility criteria are given in Supplementary Table 1 and in the original study (5). Patients were randomized to receive either *placebo* or 200 mg hydrocortisone (HC) daily for 5 days, followed by a tapering dose until day 11. Demographic, baseline and progression characteristics were extracted from the CORTICUS database.

In addition to the standard protocol, the Berlin sub-group of CORTICUS (consisting of 13 of the 52 study centers) sampled blood for subsequent measurement of cytokines and other circulating inflammatory mediators from all 84 patients. Seventy-nine out of these patients received norepinephrine at baseline while none received epinephrine. Blood samples were taken directly before an ACTH stimulation test and administration of the study medication. Supplementary Table 2 shows the timing of blood sampling relative to the onset of shock. The average time period between onset of septic shock and blood sampling was 29.4 h (standard deviation: 16.6 h). Blood samples were collected on day 0, on day 2, on the morning of day 5 (end of full dose HC application), on day 12 (day after HC cessation), day 17 and day 27. The soluble mediators interleukin (IL) 6, 8, 10, 12p70, IFNγ, TNFα, soluble TNF-receptor-I (sTNF-RI), soluble FAS (all OptEIA^™^ Set Human, BD Biosciences, New Jersey, USA), and E-selectin (R&D, Minnesota, USA) were measured in serum, plasma, or culture supernatant by enzyme-linked immunosorbent assay (ELISA) according to the manufacturers' instructions. This included calculating calibration and standard curves. All measurements were performed in duplicate. One patient was removed due to lack of cytokine data. Of the remaining 83 patients, serum lactate values (pre-treatment) and serial values were available in 53 and 41 patients, respectively. For 10 patients either IFNγ or IL10 were below the detection limit. Excluding these patients did not change the overall results. For details, see Supplementary Text 1.

The first validation set was obtained by the Hellenic Sepsis Study Group from septic shock patients with community-acquired pneumonia or intraabdominal infection. This study included a prospective collection of clinical data and biosamples from patients admitted to 45 study sites in Greece. Patients were enrolled after written informed consent provided by themselves or their legal representatives. Eligibility criteria are given in Supplementary Table 1. All enrolled patients had been reclassified into infection and sepsis using the Sepsis-3 classification criteria (15, 16). In HC-treated patients 200 mg/day HC had been administered for 7 days followed by gradual tapering. Start of administration of hydrocortisone and sampling of blood was later than 3 h after onset of septic shock. After discarding data from patients dying or being discharged on the day of admission, a total of 342 shock patients were selected. Secreted cytokines were measured using the LEGENDplex Human Inflammation Panel (13-plex, BioLegend, San Diego, USA) according to the manufacturer's instructions with half of the reagents volume and sample incubation at 4°C overnight. After removing all specimens with <8 of 13 successful cytokine measurements or without IFNγ and IL10 measurements, a total of 246 eligible shock patients (HC treatment: *n* = 93, No HC treatment: *n* = 153) were selected. If only one of the cytokines (IFNγ or IL10) was below the detection limit, the respective value was set as the detection limit. Excluding these samples from the analysis did not alter the findings (Supplementary Text 7). Propensity score matching (PSM) was not necessary as the available dataset did not consist of unbiased baseline variables. For more details and the patient characteristics see Supplementary Text 2 and Supplementary Table 3. As a further validation cohort, we investigated serum IFNγ/IL10 of patients in the *placebo* arm of the randomized and *placebo*-controlled trial of Sodium Selenite and Procalcitonin-guided antimicrobial therapy in Severe Sepsis (SISPCT) (17). SISPCT was a multicenter, randomized, clinical, 2 × 2 factorial trial performed in 33 intensive care units in Germany. It was conducted from November 6, 2009, to June 6, 2013, including a 90-day follow-up period. In this study patients were randomly assigned to receive sodium selenite or placebo. In addition, patients were randomized to receive anti-infectious therapy guided by a procalcitonin algorithm or without procalcitonin guidance. Using the same protocol as for HSSG blood samples, secreted cytokines were measured for our study using the LEGENDplex Human Inflammation Panel (13-plex, BioLegend, San Diego, USA) according to the manufacturer's instructions with half of the reagents volume and sample incubation at 4°C overnight. We selected septic shock patients from the SISPCT trial randomized to the placebo (non-selenite) treated arm with or without procalcitonin guidance. Here, administration of HC was at the discretion of the treating physician. Patients were included for our study only, if they were either not treated with HC (arm not treated with HC), or treated with at least 50 mg daily hydrocortisone for at least the first 3 days (HC treated arm). Patients were excluded if IFNγ and IL10 both were below the detection limits. If only one of these cytokines (IFNγ, IL10) was below the detection limit, the respective value was set at the detection limit. After removing all specimens with <8 out of 13 successful cytokine measurements or without IFNγ and IL10 measurements, a total of 254 eligible shock patients (HC treatment: *n* = 77, No HC treatment: *n* = 177) were selected. We observed considerable differences in their clinical variables (Supplementary Table 4a), hence we needed to select a balanced cohort employing propensity score matching (see below, Supplementary Table 4b). In addition, we analyzed the serum IFNγ/IL10 ratio of patients from an earlier small crossover study of patients in septic shock (18). Details about this study and the crossover scheme is given in Table 2g, Supplementary Text 3. In this study, the early arm got a comparable HC application as the/verum arm of CORTICUS, and was also used for validating our marker. The cytokine values of the crossover study were taken from the original publication.

### Propensity Score Matching

To account for the non-random application of hydrocortisone within the HSSG and SISPCT studies, we performed a PSM procedure using the 24 and 182 available baseline variables of these studies. According to (19), features are unbalanced if they show an absolute standardized mean difference *d* > 0.1 between HC-treated and untreated patients, and are thus candidates for PSM. Furthermore, to identify true confounding variables, we applied the method described by Austin (20) and determined features which were significantly correlated with treatment (HC) and outcome (28-days survival). Within HSSG, no variables fulfilled these criteria, and thus patient balancing using PSM was not applied. Within SISPCT, 11 out of 182 variables were significantly correlated with treatment and outcome, and showed *d* > 0.1. These variables were regarded as true confounding and comprised the variables minimal value of thrombocytes (<24 h before inclusion), distorted metabolism of glucose (yes/no), deregulated body temperature (yes/no), known location of infection (yes/no), sodium minima (<24 h before inclusion), gram positive infections (yes/no), SOFA subscore of renal function, paO_2_/FIO_2_ ratio, amount of urine, potassium maxima (<24 h before inclusion and arterial hypotension (yes/no)). This set of true confounding features was used for PSM at both leaves of IFNγ/IL10 ratio high and low using the R package matchIt (method “nearest,” caliper value of 0.2). We repeated this PSM analysis 99 times (with different random seeds) and calculated a robust significance value by calculating the median *p*-value of the corresponding Fisher's exact-tests. By counting the re-occurrence of patient numbers within 100,000 Propensity Score Matching runs, we determined the consensus cohort comprising patients with the maximal number of selections (*n* = 86,273). By this, a balanced cohort of *n* = 118 patients (HC treatment: *n* = 42, No HC treatment: *n* = 76) was determined and used for the following analysis. Supplementary Tables 7a, 7b show the patients' characteristics before and after balancing.

### *In vitro* Whole Blood Culture Experiments and *in vivo* Studies on Septic Mouse Models

To assess biological plausibility of the data-driven biomarker IFNγ/IL10, we performed *in vitro* whole blood culture experiments in which blood of healthy donors was challenged with bacterial lysates or LPS (endotoxin of *E. coli B4:O111*, Sigma Aldrich) to simulate pathogen burden. Two hundred microliter of diluted (HBSS, 1:1, V/V) heparinized whole blood obtained from healthy volunteers (male, 20–25 years, *N* = 2–5) was stimulated with serial dilutions either of LPS, lysates from two *E. coli*, two *S. aureus*, one *E. faecalis* or one *E. faecium* isolates obtained from septic patients. In addition, lysates from lab strain *S. aureus USA300* were obtained. Bacterial lysates were created by heat inactivation, following sonification and a serial dilution of the obtained fragment stock. Following incubation (37°C, 18 h, gentle agitation at 2 rpm) plasma supernatant was prepared by centrifugation (2,500 g at room temperature for 10 min). Secreted cytokine levels were measured using the LEGENDplex Human Inflammation Panel (13-plex, BioLegend), as described above. Comparison was made to vehicle controls. The data of the *in vivo* septic mouse model [cecal ligation and puncture (CLP)] for IFNγ and IL10 was provided from Dahlke et al. (21) and the log2-ratio of IFNγ and IL10 was calculated. Additionally, a second group of mice were infected by peritoneal contamination and infection (PCI). For the study, male C57BL/6J mice had been used (untreated controls: *n* = 7, sham: *n* = 3, CLP septic mice: *n* = 5, PCI septic mice: *n* = 6). Throughout all experiments, animals were kept under standardized conditions with access to food and water *ad libitum*. Cecal ligation and puncture and PCI were performed as described elsewhere (22). Whole blood was taken from CLP, PCI, sham, and control mice, for CLP, PCI, and sham mice 6 h post-intervention. For plasma preparation whole blood was centrifuged at 2,000 g for 10 min. Afterwards samples were frozen and stored at −80°C for further analysis. Plasma cytokine levels were measured using the CBA assay (Cytometric Bead Array, CBA; Mouse Inflammation Kit, Becton Dickinson, East Rutherford, USA) by FACS according to product manuals.

### Strategy for Data Analysis

We used data consisting of 137 features (patient variables, potential predictors) from 83 patients of a subcohort of the CORTICUS study population to perform an exploratory data analysis. Only data which was available at baseline was used for propensity matching and machine learning. This table also included measured cytokine levels and ratios of all possible cytokine combinations. We aimed to find a theranostic marker distinguishing HC responders (i.e., survivors) from non-responders (non-survivors). The complete list of these features is shown in Supplementary Table 5 and the workflow is depicted in Supplementary Figure 1. From this data, we sought the best predictor of 28-day survival for the placebo arm using one-level decision trees (stumps) calculated by a leave-one-out cross-validation scheme. The best stumps were chosen by enumeration of all available variables. To apply the marker to other datasets than the CORTICUS subset, the threshold was newly calculated by selecting the one with best significance of improved survival (employing a Fisher's exact-test). All analyses were carried out in R (www.r-project.org) using custom scripts.

The results from the CORTICUS, the HSSG, the SISPCT and the Crossover study cohorts were integrated by using a Mantel-Haenszel test implementing the R stats function mantelhaen.test.

## Results

### IFN*γ*/IL10 Stratifies CORTICUS Patients

The CORTICUS study included 499 patients (5). The Berlin sub-study (Immune sub-study) comprised of 84 patients, and only for these cytokine measurements were available (see section Materials and Methods). Hence, our analyses focused on this sub-cohort. Its patients' characteristics are summarized in Table 1. Median plasma concentrations of soluble mediators and leukocytes were not significantly different between the arms at baseline (Table 1). Age differed but was not a confounder (see Supplementary Text 4). Analysis of baseline characteristics was performed on 137 variables including demographic and clinical variables, Sepsis-related Organ Failure Assessment (SOFA) scores, lymphocyte counts, plasma protein concentrations of cytokines and patient blood stimulation experiments. We performed a leave-one-out cross-validation with one-level decision trees (using only one predictor at a time) to the *placebo* arm to discriminate between 28-day-survivors and non-survivors. In 95% of the cross-validation runs, the serum IFNγ/IL10 ratio (referred to as IFNγ/IL10 or ratio in the following) was selected as the best predictor with a stable threshold of 0.95. Patients in which IFNγ/IL10 was below 0.95 were denoted “low-ratio patients,” the others “high-ratio patients.” Untreated high-ratio patients showed a higher survival rate than such with a low ratio (95 vs. 45%, Table 2a). Upon applying this predictor to HC-treated patients, the reverse behavior was observed i.e., a low IFNγ/IL10 indicated a high likelihood of survival (85 vs. 69%, Table 2b).

**Table 1:** Patient characteristics, initial cytokine and blood counts of the studied CORTICUS sub-cohort.

	**HC**	**Placebo**
	**(*N* = 42, 49.4%)**	**(*N* = 41, 50.6%)**
Sex (female)	13 (31%)	11 (27%)
Age (years)	59 ± 15^*^	69 ± 11^*^
**Admission category**		
- Medical	1 (2%)	0 (0%)
- Emergency surgery	39 (93%)	37 (90%)
- Elective surgery	2 (5%)	4 (10%)
Temperature (°C)	38.5 ± 1.2	38.6 ± 0.8
Heart rate (bpm)	121 ± 18	123 ± 24
Systolic blood pressure (mmHg)	83 ± 17	90 ± 22
SOFA score at inclusion	10.4 ± 3.2	9.8 ± 2.7
SAPS II score	43.7 ± 15.6	48.0 ± 14.9
Leukocytes (thousands/mm^2^)	13.8 ± 7.3	15.1 ± 8.8
Platelets (thousands/mm^2^)	187 ± 121	165 ± 98
Arterial lactate (mmol/L)	3.0 ± 2.3	3.9 ± 4.0
**Survival**		
- Day 28 (survivors)	31 (74%)	29 (71%)
- ICU (survivors/all)	27/42 (64%)	28/41 (68%)
- Hospital (survivors/all)	25/42 (60%)	26/41 (63%)
- One year (survivors/all)	20/41 (49%)	17/40 (42%)
ICU-stay (days) all patients	28 ± 27	24 ± 19
Hospital stay (days) all patients	51 ± 42	46 ± 31
Cytokines^**^	Median (Q1–Q3)	Median (Q1–Q3)
IFNγ (pg/ml)	35.05 (15.79–152.13)	30.16 (17.93–57.80)
IL10 (pg/ml)	31.21 (13.30–76.22)	31.66 (15.68–54.76)
IL12 (pg/ml)	10.03 (3.90–43.60)	13.00 (3.90–43.27)
IL6 (pg/ml)	388.02 (177.80–487.27)	377.21 (237.17–509.01)
IL8 (pg/ml)	130.95 (56.63–226.28)	86.81 (53.81–244.44)
sFas (pg/ml)	2137.17 (1544.84–3335.26)	2155.38 (1551.38–3196.85)
sTNF-R1 (pg/ml)	25,806 (13,322–40,599)	18,809 (12,009–28,885)
White blood cell counts^**^		
Lymphocytes (/nl)	0.71 (0.34–0.93)	0.75 (0.41–1.05)
Monocytes (/nl)	0.53 (0.36–0.80)	0.56 (0.33–0.92)
NK cells (/nl)	0.04 (0.02–0.07)	0.05 (0.03–0.12)
PMNs (/nl)	11.69 (7.17–14.36)	11.06 (7.75–15.21)
B-lymphocytes (/nl)	0.09 (0.04–0.20)	0.08 (0.05–0.13)
T-Helper lymphocytes (/nl)	0.30 (0.13–0.43)	0.26 (0.14–0.50)
T-Suppressor lymphocytes (/nl)	0.10 (0.05–0.14)	0.07 (0.04–0.17)
Thrombocytes (/nl)	170.00 (99.50–250.00)	146.50 (92.00–203.25)

* *Values are given in means ± SD for all continuous variables of the patient characteristics*.

** *All cytokine and blood count values did not significantly differ between verum and placebo treated patients*.

**Table 2:** Survival rates according to high and low IFNγ/IL10.

	**Non-survivors**	**Survivors**	**% Survivors**
**(a) CORTICUS patients treated with** ***placebo***			
IFNγ/IL10 high	1	20	95% 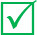 ^*^
IFNγ/IL10 low	11	9	**45%** 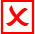
**(b) CORTICUS patients treated with verum (HC, hydrocortisone)**			
IFNγ/IL10 high	9	20	**69%** 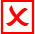
IFNγ/IL10 low	2	11	**85%** 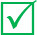
**(c) HSSG patients, not treated with HC**			
IFNγ/IL10 high	43	36	**46%** 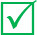
IFNγ/IL10 low	53	21	**28%** 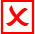
**(d) HSSG patients, treated with HC**			
IFNγ/IL10 high	31	14	**31%** 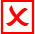
IFNγ/IL10 low	29	19	**40%** 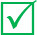
**(e) SISPCT patients, not treated with HC**			
IFNγ/IL10 high	0	15	**100%** 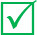
IFNγ/IL10 low	13	48	**79%** 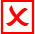
**(f) SISPCT patients, treated with HC**			
IFNγ/IL10 high	2	7	**78%** 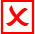
IFNγ/IL10 low	5	28	**85%** 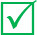
**(g) Patients from the early arm of the crossover study (treated with HC)**			
IFNγ/IL10 high	5	6	**55%** 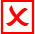
IFNγ/IL10 low	1	8	**89%** 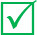

* *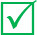; Compliant to the rule; 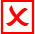 Non-compliant to the rule*.

**Figure 1:**
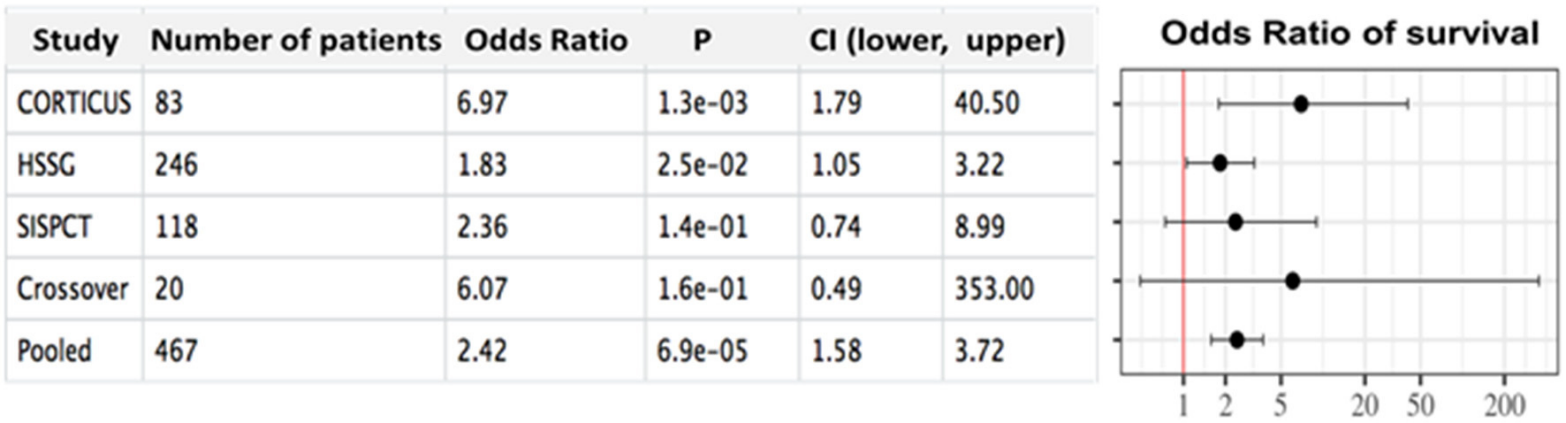
Forest plot showing the outcome in the investigated cohorts depending on hydrocortisone administration according to the theranostic marker. Odds ratios including confidence intervals and thresholds between low and high ratios are shown regarding the IFNγ/IL10 prediction rule for the sub-cohorts of CORTICUS (discovery set), HSSG (validation set), SISPCT (validation set) and the crossover study (validation set).

Performing a logistic regression with HC treatment, IFNγ/IL10 ratio and their interaction as independent variables and 28-day survival as the dependent variable, we observed a significant effect of the interaction term (*p* = 0.042, Supplementary Text 5 and Supplementary Figure 2A). These observations suggested a new decision rule, that is, no HC treatment if the ratio is high, and HC treatment if it is low. This decision rule yielded an odds ratio of survival of OR= 6.97 (95% Cl: 1.79–40.50), *p* = 0.0013 (Figure 1). Notably, the identified rule was valid also when regarding an earlier or later onset of septic shock before inclusion (0–14, 24–48, and 48–72 h, see Supplementary Figure 6F). Regarding white blood cell (WBC) counts of different leukocytes did not reveal a clear separation of IFNγ and IL10 producers. The IFNγ/IL10 ratio did not significantly correlate with any of the investigated leukocyte subtypes. Notably, IL-10 negatively correlated with most of the leukocyte subtypes (Supplementary Table 6). However, neither IFNγ, IL10 nor any WBC count alone could be used for a treatment rule.

### Validation Based on Patients From the Hellenic Sepsis Study Group, a Published Crossover Study and the “Trial of Sodium Selenite and Procalcitonin-Guided Antimicrobial Therapy in Severe Sepsis”

Supplementary Table 3 summarizes the demographics of the HSSG validation cohort. Applying IFNγ/IL10 to HSSG patients, we observed a similar pattern as for the CORTICUS subgroup (Tables 2c, d). High IFNγ/IL10 indicated distinct higher survival of the HC non-treated patients (46 vs. 28%). In contrast, in the HC treated group we observed the opposite behavior (31 vs. 40%), yielding an odds ratio of 1.83 (95% CI: 1.05–3.22), *p* = 0.025. As the means to measure the cytokines differed, we had to adjust the IFNγ/IL10 ratio threshold for the HSSG (threshold = 1.01). For the HSSG study, days of survival were available. Thus, we assembled survival curves for patients treated according and against our rule (Supplementary Figure 3). During the first 10 days, patients with a high IFNγ/IL10 ratio showed a higher rate of survival, suggesting an initial better condition of patients with higher ratios (this is evidenced also by their lactate levels, see next section). After 10 days, the HC treatment (during the first 7 days following by gradual tapering) seems to impact the outcome, as the survival rate of the low ratio patients with HC-treatment (treatment in compliance with our rule) crossed the high ratio patients with HC-treatment (treatment not in compliance with our rule). The HSSG cohort consisted of patients in sepsis either caused by community acquired pneumonia (CAP) or intra-abdominal infection. To note, our rule performed better for the subcohort of patients with intra-abdominal infection [*n* = 98 patients, OR = 2.55 (95% CI: 1.03–6.49), *p* = 0.038], compared to patients with CAP [*n* = 148 patients, OR = 1.73 (95% CI: 0.82–3.73), not significant].

Next, we analyzed serum IFNγ/IL10 of patients from an earlier, smaller crossover study (18) in which the early arm got a comparable HC application as the HC arm of CORTICUS (details about this study and the crossover scheme is given in Supplementary Text 3). In line to the results from CORTICUS and HSSG, low IFNγ/IL10 indicated better survival (89% survivors), whereas high IFN/IL10 was an indicator for considerably worse outcome (55% survivors), yielding an odds ratio of 6.07 (95% CI: 0.49-353), *p* = 0.16. Due to the small sample size the result from the crossover study failed to achieve significance.

Furthermore, we investigated serum IFNγ/IL10 of propensity score matched patients from the *placebo* arm of the randomized *placebo*-controlled trial of Sodium Selenite and Procalcitonin-guided antimicrobial therapy in Severe Sepsis (SISPCT) (17). For details about the study and the selection of patients, see Methods and Supplementary Text 6. In contrast to the former patient populations, the 28-day-survival rate of SISPCT patients receiving or not receiving HC differed considerably (68 vs. 84%, respectively, *p* = 0.0041, Fisher's exact-test, see Supplementary Figure 4A). We evaluated if there was a variable which may elucidate such an uneven distribution and identified the time of septic shock before inclusion (and HC treatment) to be strongly correlated with the survival rate of the HC-treated patients. This variable was not correlated with the survival of the non-HC-treated patients (Supplementary Figure 4B). Thus, we excluded patients with shorter time intervals between shock and study inclusion (<7 h), and balanced the remaining patients after separation into IFNγ/IL10 ratio low and high. The cohort comprised of n=118 patients and showed comparable survival rates of HC- and non-HC-treated patients (83 vs. 84%).

In summary, and comparable results when applying our treatment rule (Tables 2e,f). the investigated patients from all investigated validation studies evidenced IFNγ/IL10 as a potential theranostic marker for HC application in septic shock. Figure 1 shows the forest plot including all investigated studies.

### CORTICUS Time Courses of Serum Lactate and Norepinephrine Requirement Reflect Hemodynamic Stabilization in Patients Treated in Compliance With the Decision Rule

**Figure 2:**
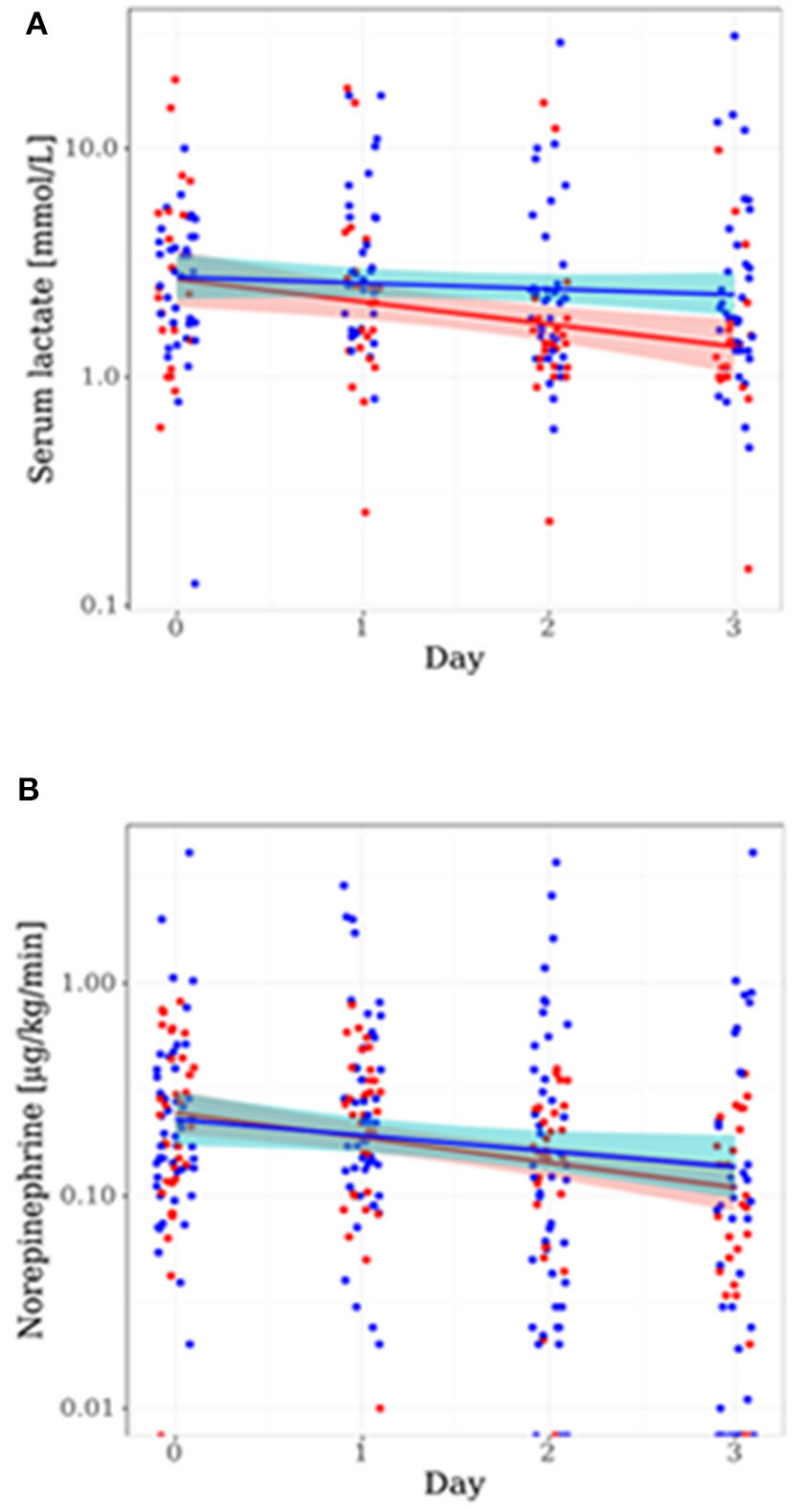
Time courses of serum lactate and norepinephrine requirement are depending on the proposed theranostic rule. Shown are patient values and linear models including color-shaded confidence intervals (95%) for the sub-populations of CORTICUS treated in compliance (red) and not in compliance (blue) to our rule. The time course is given in days after start of the study, **(A)** shows the course of serum lactate, and **(B)** shows the course of the norepinephrine requirement.

High serum lactate levels have been demonstrated to indicate severity of metabolic derangements and increased mortality in sepsis (15). In all studied subpopulations where it was available, median initial lactate was distinctly lower in patients with high compared to patients with low IFNγ/IL10 ratio (CORTICUS: 1.90 vs. 2.89 mmol/L, SISPCT: 2.20 vs. 3.00 mmol/L, Crossover study: 1.49 vs. 2.24 mmol/L). Together, lactate values were significantly different between low ratio and high ratio patients (*p* = 0.0029, *n* = 330, Yuen's bootstrap-test), suggesting that IFNγ/IL10 associated with disease severity. Of note, although the lactate levels correlate inversely with IFNγ/IL10 at baseline, serum lactate itself performed worse as a theranostic marker. Notably, we identified a significantly stronger decrease of the lactate values of patients which were treated in compliance with the decision rule in comparison to patients which were treated against the rule (Figure 2A). Hemodynamic stabilization was also supported by a visible stronger trend of reduction of norepinephrine (NE) requirement specifically in the group of patients in compliance with the decision rule (Figure 2B). In summary, time series of serum lactate and NE requirement reflect better recovery of septic shock in patients treated in compliance with the proposed theranostic marker.

### IFNγ/IL10 as an Indicator of Pathogen Challenge

**Figure 3:**
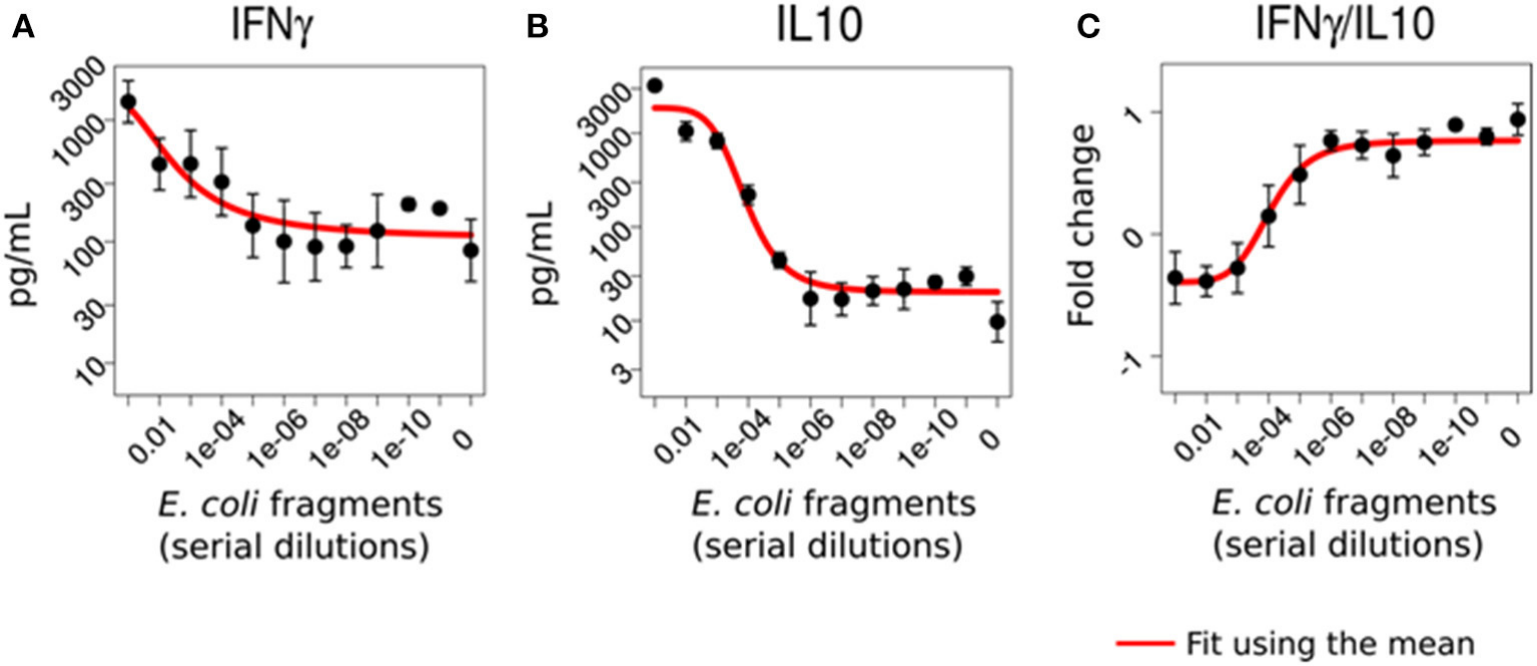
IFNγ/IL10 reflects the immunological load of immune cells *in vitro*. Whole blood from five healthy donors was challenged with varying dilutions of *E. coli* fragments mimicking the bacterial burden on the immune system in sepsis. IFNγ **(A)** and IL10 **(B)** were elevated with increasing bacterial challenge while their ratio **(C)** showed the opposite behavior, i.e., a high stimulus was associated with a lower ratio (given in log-two-fold-changes).

In light of published evidence associating IFNγ and IL10 with the severity of parasitic infection and tuberculosis (23, 24), we investigated whether IFNγ/IL10 reflects the pathogen burden of immune cells when challenged with typical pathogens found in sepsis. We spiked blood from healthy donors with fragments of clinical isolates (*Escherichia coli, Staphylococcus aureus, Enterococcus faecalis, Enterococcus faecium*) or endotoxin (LPS) across a wide range of concentrations. As expected, the higher the pathogen challenge, the higher the immune response and hence the concentration of IFNγ and IL10 in the supernatant. Remarkably, we observed the inverse behavior for the ratio, i.e., a high IFNγ/IL10 ratio for low pathogen concentrations and *vice versa*. Figure 3 shows the results exemplarily for one *E. coli* isolate. Results for the second *E. coli* isolate, *S. aureus, E. faecalis* and *E. faecium* isolates and LPS are shown in the Supplementary Material (Supplementary Figure 5). To note, testing also a lab strain of *S. aureus* (USA 300) showed not this tendency (Supplementary Figure 5, see section Discussion). To validate the obtained results in an *in vivo* sepsis model we challenged mice by cecal ligation and puncture (CLP sepsis model) as well as peritoneal contamination and infection (PCI), and compared them to untreated controls and sham treated mice (sham-operated mice underwent an identical operation except for the actual cecal ligation and puncture). Indeed, we observed the same behavior: the ratio of IFNγ/IL10 was distinctively higher in the controls (negative and sham treated mice) and lower in septic mice (Supplementary Figure 6).

Furthermore, we associated IFNγ/IL10 to the bacterial load in critical ill patients, and compared publically available data of patients with and without bacteremia. Matera et al. (25) investigated 52 patients (39 survivors and 13 non-survivors) with signs of SIRS at hospital admission. Only 4% of them were in septic shock. Twenty-eight of 52 were diagnosed with bacteremia. In line with our experimental observations, a distinctively higher ratio (1.6 ± 0.77) for IFN/IL10 in SIRS patients was observed compared to patients with bacteremia (0.8 ± 0.88, details: Supplementary Text 7). Healthy volunteers showed the highest IFNγ/IL10 ratio (2.80 ± 1.22) of all groups investigated by Matera et al.

In summary, IFNγ/IL10 inversely correlates with the immunological load of infection in an *in vitro* system, in septic mice and when comparing bacteremic and non-bacteremic critically ill patients.

## Discussion

Starting from a larger panel of 137 predictors derived from septic shock patients (*n* = 83) in CORTICUS, we identified the ratio of serum IFNγ and IL10 as a promising biomarker to guide the treatment decision for or against HC. We validated our results by applying the marker to three other datasets.

Metabolically, high serum lactate indicates severity of cellular disturbances (15) and is associated with poor outcome (26). Consistent with this, patients with low IFNγ/IL10 showed high serum lactate. Furthermore, stratifying patients in compliance with the proposed decision rule, we observed a considerable decrease over time in serum lactate specifically in the group of patients, which were treated in compliance with the theranostic marker. This further extends the concept that HC is generally able to stabilize cardiovascular function in septic shock and suggests that this effect might also be restricted to patients with a high burden of the disease, and compares to the different beneficial effects of steroids in septic shock observed in the trials mentioned in the introduction (French studies with higher mortality showed a beneficial effect compared to no beneficial effect in the studies ADRENAL and CORTICUS).

IFNγ and IL10 have been suggested as biomarkers for parasitic (23) and other infections, such as tuberculosis (24). E.g. McIlleron et al. reported the time course of IFNγ indicating response to treatment in pulmonary tuberculosis (27). However, for diagnosis of pulmonary tuberculosis, IFNγ alone has limitations relating to sensitivity (28). In turn, high IL10 was observed with mycobacterial persistence (29) suggesting that in particular the ratio of these cytokines might reflect the immunological burden of infection. In line with this, it has been shown that the ratio of IFNγ and IL10 correlates with disease severity of tuberculosis and may differentiate pulmonary from extra-pulmonary forms (30). To elucidate the functional implications of low and high IFNγ/IL10, we performed blood culture assays challenging the immune cells with fragments of bacterial isolates from septic patients. Indeed, we observed the ratio to be negatively correlated with the load of pathogen fragments. Similarly, we observed a significant lower ratio in untreated and sham controls compared to septic mice. This was supported by clinical data from Matera et al. (25) when regarding critically ill patients with bacteraemia (higher bacterial load) and without bacteraemia (no or non-detectable bacterial load). Thus, IFNγ/IL10 is supposedly (negatively) associated with the pathogen load.

IFNγ has been consistently documented to activate cells of adaptive immunity (31), and IL10 as a suppressor of innate immune responses and inflammation (32, 33). Hence, high IL-10 may be a response to a high load to prevent and limit over-whelming immune reactions and, in consequence, tissue damage, whereas low IFNγ reflects a low adaptive immune response.

Interestingly, Sommerfeld et al. observed recently that mouse mutants lacking the receptor of complement factor C5a showed increased survival in mild septic mouse models, and, IFNγ/IL10 was increased compared to wildtype mice under the same conditions (34). The mutants also showed a similar survival benefit when mutant and wildtype mice were treated with antibiotics. Sommerfeld et al. explained the effect by an increased adaptive immune response in the mutants. It is reasonable that a high pathogen load causes repression or even disruption of the adaptive immune system or/and induces an overwhelming innate immune response monitored by the ratio of IFNγ/IL10. Nevertheless, this needs exploration in future studies. We observed *in vitro, in vivo* and in patients that blood challenged with bacteria/bacterial fragments displayed lower IFNγ/IL10 at higher loads, and *vice versa*. Transferring these observations to septic shock patients with low IFNγ/IL10, we speculate that HC treatment yielded a better outcome in these HC-responders because HC may allow the highly loaded immune system more pace for recovery. This is evidenced by the reduction of serum lactate and norepinephrine requirements as measures of recovery particularly in the subgroup of very sick patients.

To mention, we also challenged the blood culture with the lab strain *S. aureus* USA300, for which this behavior was not observed. We speculate that such a pathogen causing rather skin and soft tissue inflammation than bacteraemia and sepsis may evoke other cytokine responses and suggest follow up analyses on this aspect in more detail.

The concern about side effects of corticosteroids such as infections in patients with less severe septic shock stipulated more restrictive recommendations by the Surviving Sepsis Campaign. HC application is currently recommended only for patients not responding to adequate fluid resuscitation and vasopressor therapy (35, 36). Considering an immune biomarker, such as IFNγ/IL10 reflecting the status of the patients' immune system status may intuitively be a promising path to improve the treatment decision. Consistent with this concept, Bentzer et al. studied data from patients of the “VAsopressin vs. norepinephrine in Septic Shock Trial” (VASST) investigating corticosteroid treated vs. non-treated patients (only vasopressin or catecholamine vasopressors). They identified a signature of three cytokines (IL3, IL6, CCL4) suggesting response to corticosteroid treatment (37). However, these results based on a study, which was not randomized, blinded or protocolized according to corticosteroid treatment, and Bentzer et al. did not elaborate how these cytokines interact with corticosteroid treatment.

In a very recent randomized controlled trial, patients who were hospitalized with Covid-19 were randomly assigned to receive dexamethasone (RECOVERY) or placebo. This trial elucidated that dexamethasone treatment is beneficial for patients receiving either invasive mechanical ventilation or oxygen alone at randomization but not among those receiving no respiratory support (38). Here the supportive measurement of the affected organ was sufficient for predictive enrichment of steroids treatment. In our case (septic shock), predictive enrichment basing solely on the supportive measurement (vasopressor requirement) was not sufficient and we needed to monitor the immune status (by the cytokines IFNγ and IL10). As an intriguing project for the future, we propose developing a generic rule distinguishing different disease/syndrome entities employing such categories.

### Limitations and Strengths

We excluded patients from the very early window of septic shock of SISPCT (<7 h) to balance the survival between HC-treated and HC-untreated patients. This difference was not surprising, as the HC treatment was applied to patients in more severe conditions, as suggested by guidelines. This is consistent with the CORTICUS data where inclusion criteria were broader (up to 72 h) and the onset of sepsis in CORTICUS occurred on average 29 h before inclusion. In the light of the current recommendations to use HC restrictively, it might indicate, that HC treatment could be supported by a theragnostic biomarker. In addition, we were not able to outbalance every variable in SISPCT. Another limitation of our study is the variability of thresholds to distinguish between high and low IFNγ/IL10 ratio. For CORTICUS it was 0.95, for HSSG 1.01, for SISPCT 0.48, and for the crossover study it was 0.81. DeJager et al. (33) reported that timing of sampling, sample storage, the choice of the platform and the blood collection tubes are crucial issues when measuring cytokines from blood. Cytokines can be stored below −80°C for a longer time, but even at −80°C degradation was observed, in particular of IL10. Most of these parameters varied between the different studies that we included in our analysis making it difficult to compare the ratios between the studies. Our cytokine measurements seemed to depend on the assay and sample handling described by DeJager et al. The discrepancies of the optimal threshold between the studies may also be due to the rather older time point when Corticus and the Crosscover study were performed. Hence, when implementing the rule into the clinics, well defined standard operating procedures need to be developed to obtain an appropriate, center independent threshold. A limitation of the *in vitro* blood culture study is that we used only blood of younger male volunteers.

A strength of our study is that our rule seems to be valid across studies of very heterogenous survival rates (CORTICUS: 72%, Crossover: 70%, HSSG: 37%, SISPCT: 84%). Applying our decision rule successfully across a broad range of these studies suggests the marker to be generic. The analyzed Berlin cohort of CORTICUS contained a high number of surgical patients and a rather low number of medical patients. Also the averaged ICU and hospital stay was longer as usual for septic shock patients [when compared e.g. to the prospective INSEP study (39)]. The validation cohorts didn't show this notable discrepancy suggesting a good generalizability of our rule. A randomized controlled trial also better representing these variables is, thus, in every case inevitable to confirm the identified rule. Furthermore, strengths of our study relates to the use of (i) well-phenotyped cohorts, (ii) that our marker showed remarkably similar results across all studies, and (iii) that we provide clinical, *in vivo* and *in vitro* data relating it to the pathogen challenge of the immune system.

## Conclusions

We identified the ratio of serum cytokines IFNγ and IL10 as a potential theranostic marker for hydrocortisone treatment in septic shock. An accompanying study into the mechanism suggests that this ratio monitors the immune response challenged across distinct pathogen loads. The development of a standard operating procedure for obtaining the cytokine concentrations in serum followed by a clinical trial is necessary confirming our rule before implementing it into the clinical routine.

## Data Availability Statement

The original contributions generated in the study are included in the article/Supplementary Material, further inquiries can be directed to the corresponding author/s.

## Ethics Statement

The CORTICUS trial was a multicenter study, the protocol was approved by the Ethics Committee at each of the 52 participating intensive care units. In addition to the standard CORTICUS protocol, the Berlin study group sampled blood for subsequent measurement of cytokines and other circulating inflammatory mediators. This was approved by the Local Ethics Committee (No: 153/2001). Written informed consent was obtained from patients, proxies or their legal representatives. The HSSG cohort represents a prospective collection of clinical data and biosamples in 45 study sites in Greece. The study protocol was approved from the Ethics Committees of all participating hospitals. Patients were enrolled after written informed consent provided by themselves or by first-degree relatives if patients were unable to consent. The Placebo-Controlled Trial of Sodium Selenite and Procalcitonin Guided Antimicrobial Therapy in Severe Sepsis (SISPCT) was a multicenter clinical trial. It was conducted in 33 multidisciplinary intensive care units across Germany. The study protocol was approved by the ethics board of Jena University Hospital. Written informed consent was obtained from all patients or their legal representatives. The study protocol for the crossover study was approved by the institutional (Charite, Berlin) Ethics Committee. All animal experiments were in accordance with the German legislation on protection of animals and with permission of the regional animal welfare committee.

## Author Contributions

RK, AK, MOS, DK, and MB conceptualized and designed the study. MOS, RK, AK, and VK developed the methodology. AK, VK, MB, MS, JB, DK, DR, and RK analyzed and interpreted the data. Animal experiments were performed by OS. Administrative, technical, or material support was given by OA, DR, GD, IT, EA, HB, ML, CS, MS, FB, MOP, HG, RC, SC, and JB. The study was supervised by RK, DK, and MB, and they are the garantor of the article. All authors read and approved the final manuscript.

## Conflict of Interest

The authors declare that the research was conducted in the absence of any commercial or financial relationships that could be construed as a potential conflict of interest.

## Abbreviations

ACTH: Adrenocorticotropic hormone
ADRENAL: Adjunctive corticosteroid treatment in critically ill patients with septic shock
CI: Confidence interval
CORTICUS: Corticosteroid therapy of septic shock
HC: Hydrocortisone
HSSG: Hellenic sepsis study group
NE: Norepinephrine
PSM: Propensity score matching
OR: Odds ratio
SISPCT: Sodium selenite and procalcitonin guided antimicrobial therapy in severe sepsis
SOFA: Sequential organ failure assessment
VASST: Vasopressin and septic shock trial.

## References

[1] Annane D Bellissant E Bollaert PE Briegel J Confalonieri M De Gaudio R. Corticosteroids in the treatment of severe sepsis and septic shock in adults: a systematic review. JAMA. (2009) 301:2362–75. 10.1001/jama.2009.815 19509383

[2] Venkatesh B Finfer S Cohen J Rajbhandari D Arabi Y Bellomo R. Investigators and G. the Australian-New Zealand Intensive Care Society clinical trials: adjunctive glucocorticoid therapy in patients with septic shock. N Engl J Med. (2018) 378:797–808. 10.1056/NEJMoa1705835 29347874

[3] Annane D Sebille V Charpentier C Bollaert PE Francois B Korach JM. Effect of treatment with low doses of hydrocortisone and fludrocortisone on mortality in patients with septic shock. JAMA. (2002) 288:862–71. 10.1001/jama.288.7.862 12186604

[4] Annane D Renault A Brun-Buisson C Megarbane B Quenot JP Siami S. Hydrocortisone plus fludrocortisone for adults with septic shock. N Engl J Med. (2018) 378:809–18. 10.1056/NEJMoa1705716 29490185

[5] Sprung CL Annane D Keh D Moreno R Singer M Freivogel K. Hydrocortisone therapy for patients with septic shock. N Engl J Med. (2008) 358:111–24. 10.1056/NEJMoa071366 18184957

[6] Hotchkiss RS Monneret G Payen D. Sepsis-induced immunosuppression: from cellular dysfunctions to immunotherapy. Nat Rev Immunol. (2013) 13:862–74. 10.1038/nri3552 24232462 PMC4077177

[7] Bauer M Giamarellos-Bourboulis EJ Kortgen A Moller E Felsmann K Cavaillon JM. A transcriptomic biomarker to quantify systemic inflammation in sepsis—a prospective multicenter phase II diagnostic study. EBioMedicine. (2016) 6:114–25. 10.1016/j.ebiom.2016.03.006 27211554 PMC4856796

[8] Kaufmann SHE Dorhoi A Hotchkiss RS Bartenschlager R. Host-directed therapies for bacterial and viral infections. Nat Rev Drug Discov. (2018) 17:35–56. 10.1038/nrd.2017.162 28935918 PMC7097079

[9] Baschant U Culemann S Tuckermann J. Molecular determinants of glucocorticoid actions in inflammatory joint diseases. Mol Cell Endocrinol. (2013) 380:108–18. 10.1016/j.mce.2013.06.009 23769823

[10] Chinenov Y Rogatsky I. Glucocorticoids and the innate immune system: crosstalk with the toll-like receptor signaling network. Mol Cell Endocrinol. (2007) 275:30–42. 10.1016/j.mce.2007.04.014 17576036

[11] Galon J Franchimont D Hiroi N Frey G Boettner A Ehrhart-Bornstein M. Gene profiling reveals unknown enhancing and suppressive actions of glucocorticoids on immune cells. FASEB J. (2002) 16:61–71. 10.1096/fj.01-0245com 11772937

[12] Busillo JM Azzam KM Cidlowski JA. Glucocorticoids sensitize the innate immune system through regulation of the NLRP3 inflammasome. J Biol Chem. (2011) 286:38703–13. 10.1074/jbc.M111.275370 21940629 PMC3207479

[13] Dhabhar FS. Stress-induced augmentation of immune function–the role of stress hormones, leukocyte trafficking, and cytokines. Brain Behav Immun. (2002) 16:785–98. 10.1016/S0889-1591(02)00036-3 12480507

[14] Cruz-Topete D Cidlowski JA. One hormone, two actions: anti- and pro-inflammatory effects of glucocorticoids. Neuroimmunomodulation. (2015) 22:20–32. 10.1159/000362724 25227506 PMC4243162

[15] Singer M Deutschman CS Seymour CW Shankar-Hari M Annane D Bauer M. The third international consensus definitions for sepsis and septic shock (sepsis-3). JAMA. (2016) 315:801–10. 10.1001/jama.2016.0287 26903338 PMC4968574

[16] Giamarellos-Bourboulis EJ Tsaganos T Tsangaris I Lada M Routsi C Sinapidis D. Validation of the new Sepsis-3 definitions: proposal for improvement in early risk identification. Clin Microbiol Infect. (2017) 23:104–9. 10.1016/j.cmi.2016.11.003 27856268

[17] Bloos F Trips E Nierhaus A Briegel J Heyland DK Jaschinski U. Effect of sodium selenite administration and procalcitonin-guided therapy on mortality in patients with severe sepsis or septic shock: a randomized clinical trial. JAMA Intern Med. (2016) 176:1266–76. 10.1001/jamainternmed.2016.2514 27428731

[18] Keh D Boehnke T Weber-Cartens S Schulz C Ahlers O Bercker S. Immunologic and hemodynamic effects of “low-dose” hydrocortisone in septic shock: a double-blind, randomized, placebo-controlled, crossover study. Am J Respir Crit Care Med. (2003) 167:512–20. 10.1164/rccm.200205-446OC 12426230

[19] Staffa SJ Zurakowski D. Five steps to successfully implement and evaluate propensity score matching in clinical research studies. Anesth Analg. (2018) 127:1066–73. 10.1213/ANE.0000000000002787 29324498

[20] Austin PC. An introduction to propensity score methods for reducing the effects of confounding in observational studies. Multivariate Behav Res. (2011) 46:399–424. 10.1080/00273171.2011.568786 21818162 PMC3144483

[21] Dahlke K Wrann CD Sommerfeld O Sossdorf M Recknagel P Sachse S. Distinct different contributions of the alternative and classical complement activation pathway for the innate host response during sepsis. J Immunol. (2011) 186:3066–75. 10.4049/jimmunol.1002741 21263075 PMC3512104

[22] Rittirsch D Huber-Lang MS Flierl MA Ward PA. Immunodesign of experimental sepsis by cecal ligation and puncture. Nat Protoc. (2009) 4:31–6. 10.1038/nprot.2008.214 19131954 PMC2754226

[23] Medina TS Costa SP Oliveira MD Ventura AM Souza JM Gomes TF. Increased interleukin-10 and interferon-gamma levels in *Plasmodium vivax* malaria suggest a reciprocal regulation which is not altered by IL-10 gene promoter polymorphism. Malar J. (2011) 10:264. 10.1186/1475-2875-10-264 21917128 PMC3196927

[24] Djoba Siawaya JF Beyers N van Helden P Walzl G. Differential cytokine secretion and early treatment response in patients with pulmonary tuberculosis. Clin Exp Immunol. (2009) 156:69–77. 10.1111/j.1365-2249.2009.03875.x 19196252 PMC2673743

[25] Matera G Puccio R Giancotti A Quirino A Pulicari MC Zicca E. Impact of interleukin-10, soluble CD25 and interferon-gamma on the prognosis and early diagnosis of bacteremic systemic inflammatory response syndrome: a prospective observational study. Crit Care. (2013) 17:R64. 10.1186/cc12596 23561467 PMC4056318

[26] Casserly B Phillips GS Schorr C Dellinger RP Townsend SR Osborn TM. Lactate measurements in sepsis-induced tissue hypoperfusion: results from the surviving sepsis campaign database. Crit Care Med. (2015) 43:567–73. 10.1097/CCM.0000000000000742 25479113

[27] McIlleron H Watkins ML Folb PI Ress SR Wilkinson RJ. Rifampin levels, interferon-gamma release and outcome in complicated pulmonary tuberculosis. Tuberculosis (Edinb). (2007) 87:557–64. 10.1016/j.tube.2007.08.002 17890156

[28] Chegou NN Sutherland JS Namuganga AR Corstjens PL Geluk A Gebremichael G. Africa-wide evaluation of host biomarkers in QuantiFERON supernatants for the diagnosis of pulmonary tuberculosis. Sci Rep. (2018) 8:2675. 10.1038/s41598-018-20855-7 29422548 PMC5805775

[29] Murray PJ Wang L Onufryk C Tepper RI Young RA. T cell-derived IL-10 antagonizes macrophage function in mycobacterial infection. J Immunol. (1997) 158:315–21. 8977205

[30] Jamil B Shahid F Hasan Z Nasir N Razzaki T Dawood G. Interferon gamma/IL10 ratio defines the disease severity in pulmonary and extra pulmonary tuberculosis. Tuberculosis (Edinb). (2007) 87:279–87. 10.1016/j.tube.2007.03.004 17532265

[31] Schoenborn JR Wilson CB. Regulation of interferon-gamma during innate and adaptive immune responses. Adv Immunol. (2007) 96:41–101. 10.1016/S0065-2776(07)96002-2 17981204

[32] Sabat R Grutz G Warszawska K Kirsch S Witte E Wolk K. Biology of interleukin-10. Cytokine Growth Factor Rev. (2010) 21:331–44. 10.1016/j.cytogfr.2010.09.002 21115385

[33] Kuhn R Lohler J Rennick D Rajewsky K Muller W. Interleukin-10-deficient mice develop chronic enterocolitis. Cell. (1993) 75:263–74. 10.1016/0092-8674(93)80068-P 8402911

[34] Sommerfeld O Medyukhina A Neugebauer S Ghait M Ulferts S Lupp A. Targeting complement C5a receptor 1 for the treatment of immunosuppression in sepsis. Mol Ther. (2021) 29:338–46. 10.1016/j.ymthe.2020.09.008 32966769 PMC7791006

[35] Dellinger RP Levy MM Carlet JM Bion J Parker MM Jaeschke R. Critical Care: surviving sepsis campaign: international guidelines for management of severe sepsis and septic shock: 2008. Crit Care Med. (2008) 36:296–327. 10.1097/01.CCM.0000298158.12101.41 18158437

[36] Dellinger RP Levy MM Rhodes A Annane D Gerlach H Opal SM. Surviving sepsis campaign guidelines committee including the pediatric: surviving sepsis campaign: international guidelines for management of severe sepsis and septic shock: 2012. Crit Care Med. (2013) 41:580–637. 10.1097/CCM.0b013e31827e83af 23353941

[37] Bentzer P Fjell C Walley KR Boyd J Russell JA. Plasma cytokine levels predict response to corticosteroids in septic shock. Intensive Care Med. (2016) 42:1970–9. 10.1007/s00134-016-4338-z 27071387

[38] Recovery Collaborative Group Horby P Lim WS Emberson JR Mafham M Bell JL. Dexamethasone in hospitalized patients with Covid-19—preliminary report. N Engl J Med. (2020) 10.1056/NEJMoa2021436. [Epub ahead of print]. 32678530 PMC7383595

[39] SepNet Critical Care Trials Group. Incidence of severe sepsis and septic shock in German intensive care units: the prospective, multicentre INSEP study. Intensive Care Med. (2016) 42:1980–9. 10.1007/s00134-016-4504-3 27686355

[40] Koenig R Kolte A Ahlers O Oswald M Röll D Dimopoulos G. Use of IFNγ/IL10 ratio for stratification of hydrocortisone therapy in patients with septic shock. BioRxiv. 10.1101/502864. [Epub ahead of print]. PMC7985546 33767693

