# Supplementary material for: Use of IFNγ/IL10 Ratio for Stratification of Hydrocortisone Therapy in Patients With Septic Shock
Source: Front Immunol. 2021 Mar 9;12:607217. doi: 10.3389/fimmu.2021.607217 (PMC7985546; doi:10.3389/fimmu.2021.607217)
Supplement: Supplementary file 1 [file Data_Sheet_1.pdf]

Supplementary Material

Content

Content....2
Supplementary Text....3
Supplementary Text 1. Blood samples and serum assays of the CORTICUS Berlin sub-cohort....3
Supplementary Text 2. The Hellenic Sepsis Study Group (HSSG) cohort....3
Supplementary Text 3. The crossover study....4
Supplementary Text 4. Age is not a confounder....4
Supplementary Text 5. Interaction analysis of treatment and biomarker....5
Supplementary Text 6. The SISPCT trial....5
Supplementary Text 7. Relating IFNγ/IL10 to SIRS patients with and without bacteremia....5
Supplementary Figures....6
Figure S1. Workflow....6
Figure S2. Interaction plots of treatment and IFNγ/IL10 and of duration of septic shock....7
Figure S3. Change of survival rates of HSSG patients after HC treatment.....8
Figure S4. Dependence of the survival rates of HC-treated SISPCT patients on the time interval between the beginning of shock and inclusion into the study....9
Figure S5. IFNγ, IL10 and IFNγ/IL10 of immune cells challenged *in vitro* with bacterial fragments or LPS 12
Figure S6. IFNγ/IL10 in septic mice and controls....12
Supplementary Tables....13
Table S1. Detailed inclusion and exclusion criteria of CORTICUS, HSSG, SISPCT and the crossover study....13
Table S2. Duration between the onset of shock and the blood drawings in the CORTICUS sub-cohort....15
Table S4. Patient characteristics of the analysed SISPCT patients....16
Table S5. List of all 137 predictors....18
Table S6. Pearson correlation of leukocyte counts to IFNγ, IL10 and IFNγ/IL10 ratio....19
Table S7. The lactate measurements available for the CORTICUS patients....19
Table S8. Patient characteristics of the analysed Crossover patients....21
Table S9. Survival rates according to high and low IFNγ/IL10, restricted to patients with IFNγ or IL10 values within the detection limits....21
References....22

Supplementary Text

Supplementary Text 1. Blood samples and serum assays of the CORTICUS Berlin sub-cohort

Blood samples were collected on day 0 (before the corticotropin test), on day 2, on the morning of day 5 (end of full dose hydrocortisone (HC) application), on day 12 (day after HC cessation), on days 17 and on day 27. The short corticotropin test was performed immediately before study drug application using blood samples taken before and 60 minutes after an intravenous bolus of 0.25 mg cosyntropin (Novartis). Blood samples were stored at 4°C for three hours to avoid time imbalances between blood collection at different sites and further processing. Serum and plasma was stored at -80°C until further analysis. Heparinized and EDTA whole blood samples were used for functional assays. At the time of the CORTICUS study, soluble mediators, interleukin-(IL)-6, -8, -10, -12p70, interferon-γ, (IFNγ), tumor necrosis factor α (TNFa), soluble TNF-receptor I (sTNF-RI), soluble FAS (all BD Biosciences OptEIA™, Set Human), and E-selectin (R&D) were measured in serum, plasma, or culture supernatant with enzyme-linked immunosorbent assay (ELISA) according to the manufacturer's instructions. This included calculating calibration and standard curves. All measurements were done in duplicate. Calculating a variation coefficient (CV) was not part of the product description. For the cytokine measurement EDTA plasma was used. Hydrocortisol was measured from plasma. Surface antigens of leukocytes were measured using flow cytometry, leukocytes in EDTA, thrombocytes in citrate plasma (platelet enriched), caspase/BCL2 leukocytes with heparin. Serum lactate was measured by routine blood gas analytics for 51 patients at day 0. Among these, 41 patients were further observed daily for 3 days (Table S7).

Restricting the analysis to patients with IFNγ and IL10 values being within the detection limits: We inspected if patients with undetectable low IFNγ or IL10 values may confound our analysis. For CORTICUS (detection limit for IFNγ: 2.35 pg/ml, for IL10: 3.90 pg/ml), 73 patients remained after removing patients with such values. For HSSG (detection limit for IFNγ: 2.94 pg/ml, for IL10: 1.10 pg/ml), 213 patients remained. For SISPCT (detection limit for IFNγ: 2.00 pg/ml, for IL10: 1.34 pg/ml), 94 patients were left. For the crossover study, all 20 patients were within the detection limits for IFNγ and IL10. Overall, the pooled data set reduced to 400 patients. Results corresponding to these restricted sets of patients are given in Table S9.

Supplementary Text 2. The Hellenic Sepsis Study Group (HSSG) cohort

The HSSG cohort represents a prospective collection of clinical data and biosamples since 2006 of patients with documented infection and at least two signs of the systemic inflammatory response syndrome (SIRS) in 45 study sites in Greece. The study protocol was approved from the Ethics Committees of all participating hospitals. Patients were enrolled after written informed consent provided by themselves or by first-degree relatives if patients were unable to consent. Patients infected by the human immunodeficiency virus, with less than 1,000 neutrophils/mm^3 and with systemic intake of more than 0.3 mg/kg of equivalent prednisone during the last 15 days before baseline were excluded. Peripheral blood was drawn within the first 24 hours of the advent of signs of SIRS after puncture of one peripheral vein under aseptic conditions. Blood was centrifuged and serum was shipped to the central lab located at the 4th Department of Internal Medicine of ATTIKON University Hospital. All enrolled patients were reclassified into infection and sepsis in

2017 using the Sepsis-3 classification criteria (2, 3). Clinical data was recorded into one case report form (CRF) that was monitored by an independent monitor. Collected information consisted of demographics, type of infection, severity scores, biochemistry, whole blood cell counting, blood gases, microbiology, administered antibiotics, medical therapy other than antibiotics, interventions and 28-day outcome. Those treated with HC received 50 mg intravenously four times daily for six days followed by gradually tapering off.

Among available patients and biosamples in the cohort, 342 with community-acquired pneumonia or intraabdominal infections and with septic shock were randomly selected and analyzed. Secreted cytokines were measured using the LEGENDplex Human Inflammation Panel (13-plex, BioLegend) according to manufacturer´s protocol with half of the reagents volume and sample incubation time at 4°C over night. After removing all specimens with less than 8 of 13 successful cytokine measurements or without IFNγ and IL10 measurements, a total of 246 eligible shock patients (HC treatment: n = 93, No HC treatment: n = 153) were selected. If only one of the cytokines (IFNγ or IL10) was below the detection limit, the value of the detection limit was taken. Excluding these samples from the analysis did not alter the findings (Supplementary Text S1). Table S3 shows the patient characteristics.

Supplementary Text 3. The crossover study

The study was published elsewhere (6). Briefly, a double-blinded, randomized, *placebo*-controlled, crossover study was performed with 40 patients diagnosed with septic shock. Until day 3, one arm received first 100 mg of HC as a loading dose and 10 mg per hour until day 3 (n = 20), followed by 3 days *placebo*. The patients treated at day 0 to day 2 (early) were treated comparably to the CORTICUS *verum* arm (CORTICUS: treated for 5 days directly at start) and were used for our study. The other arm received the first three days *placebo* (n = 20), followed by *HC* until day 6. To exclude the time point of HC application as an additional variable, we did not regard these lately treated patients as they were neither similarly treated as *placebo* nor as *verum*. Characteristics of the used 20 early treated patients are given in Table S8.

Blood samples were collected on day 0 (before randomization) and every subsequent day until day 6. At the time of the study, serum cortisol was measured with solid-phase radioimmunoassay (Biermann, Bad Nauheim, Germany). Enzyme-linked immunosorbent assays (ELISA) were used for measurement of interleukin 4, 8, 10, 12p70, and IFNγ (BD PharMingen, Germany), soluble E-selectin (BenderMed Alexis, Austria), IL6 (R&D, Wiesbaden, Germany) and of soluble tumor necrosis factor receptors I and II (Biosource, Germany). The study protocol for this study was approved by the institutional ethics committee.

Supplementary Text 4. Age is not a confounder

The average age of the HC patients of the CORTICUS subgroup was 59.4 years while for the *placebo* patients it was 69.4 years. Therefore, we repeated the analysis 1000 times using PSM (see Materials and Methods) for the feature age. We yielded a median odds ratio of 10.07 (95% CI: 1.92 – 103.25, $p = 0.0024$) of survival when applying to our rule.

Supplementary Text 5. Interaction analysis of treatment and biomarker

We carried out logistic regressions using the function glm in R based on the CORTICUS, the HSSG, the SISPCT and the pooled population. HC treatment, the IFNγ/IL10 ratio and their interaction were used as independent variables, while 28-day survival was the dependent variable. In all four patient populations, the best logistic regression model according to the Akaike Information Criteria (AIC) was a model which contained at least the interaction variable of treatment and ratio as a negative predictor of survival, which is consistent with the fact that higher IFNγ/IL10 ratios suggest no HC treatment. This logistic regression model was in the discovery set (CORTICUS, $n = 83$) significantly better than the null model ($p = 0.042$), for the other studies, it was not significant, but showed the right tendency. All interaction plots are shown in Figure S2. For the Crossover study, this analysis could not be done due to the lack of one arm.

Supplementary Text 6. The SISPCT trial

The *placebo*-controlled, randomized trial of Sodium Selenite and Procalcitonin guided antimicrobial therapy in Severe Sepsis (SISPCT) was performed in 33 intensive care units in Germany. The purpose of this study was to determine whether the intravenous application of sodium-selenite can reduce mortality in patients with severe sepsis or septic shock. Additionally, it was investigated, whether the measurement of procalcitonin - a marker of infection - can be used to guide antimicrobial therapy during the disease course. Between November 2009 and March 2013, 8,174 patients with septic shock or severe sepsis were screened and 1,089 eligible patients with informed consent were randomized.

Secreted cytokine levels in blood serum samples were determined for 1040 patients by using the LEGENDplex Human Inflammation Panel (13-plex, BioLegend) according to manufacturer´s protocol with half of the reagents volume and sample incubation time at 4°C overnight. For our study, we excluded the selenium treated patients and patients without septic shock, with high dosages of steroids, immune suppression by drugs or without survival information after 28 days. As hydrocortisone was given according to individual treatment decisions, we have to group by ourself the remaining patients according to their treatment status. As the daily dosis of HC lay between 0 and 602 mg, we selected patients with at least 50 mg daily during the first three days as HC-treated ones. We selected patients as untreated if the daily HC dosis was 0 during at least the first five days. All other patients were excluded from the analysis. We end up with 254 Patients (HC treatment: $n = 77$, No HC treatment: $n = 177$). Patient characteristics are given in Table S4a.

Supplementary Text 7. Relating IFNγ/IL10 to SIRS patients with and without bacteremia

Matera and coworkers investigated cytokine concentrations of 52 patients with diagnosis of systemic inflammatory response syndrome (SIRS) at hospital admission, of which 28 were bacteremic. Two patients had septic shock, 13 were non-survivors, 39 survivors. SIRS was defined as two or more of the criteria 1) hypothermia or fever, 2) tachycardia; 3) tachypnea, and 4) leukocytosis, leukopenia or immature band forms (for details, see (7)). Subjects under the age of 18 and patients treated with immunosuppressive drugs were excluded from the study. We obtained their cytokine measures from their article and calculated the IFNγ/IL10 ratio including the estimated error according to the used IFNγ and IL10 concentrations implementing the Gaussian error propagation law, that is, for Δy being the error of $y(x_1, x_2)$ = IFNγ/IL10 and x_1=IFNγ, x_2= IL10.

Supplementary Figures

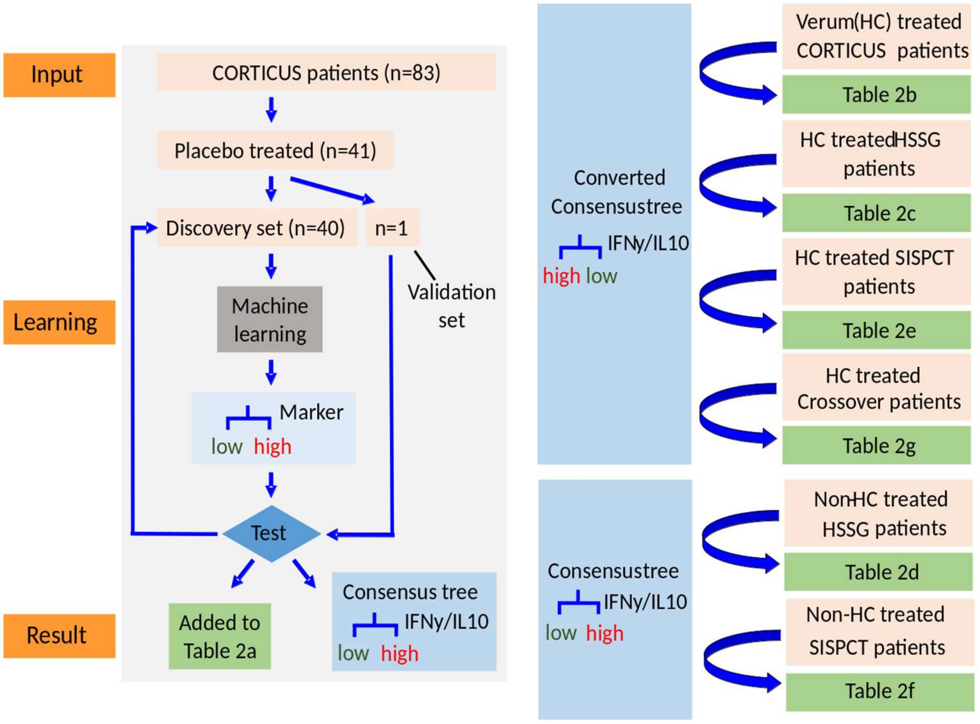

a) b)

Figure S1. Workflow

a) The algorithm for the discovery of the theranostic marker, note that here "validation set" stands for one of the CORTICUS placebo patients and not for other studies:

1) From all investigated CORTICUS patients, the placebo-treated patients are selected.

2) The selected patients are split into a training set (n = 40 and a validation set (n = 1).

3) Machine learning: Selection of the best predictor out of 137 available predictors to predict survival on the training set, using one-predictor-based decision trees.

4) Testing the performance of the selected predictor on the validation set.

5) Adding the result from 4) to the confusion matrix, and storing the tree.

6) Going back to 2). In 2) the next patient is forming the validation set, and the rest of placebo patients are the training set

7) From all stored trees, a consensus tree is determined (the one which has been used most often, i.e. high IFNγ/IL10 predicts survival, low IFNγ/IL10 predicts non-survival)

b) The converted consensus tree (low IFNγ/IL10 predicts survival, high IFNγ/IL10 predicts non-survival) is applied to the HC-treated patients of CORTICUS, HSSG, SISPCT, and to the early arm of the crossover study. The consensus tree is applied to the non-HC treated patients of HSSG and SISPCT.

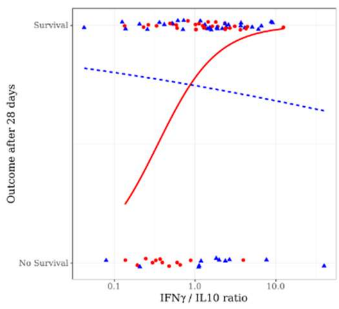

a)

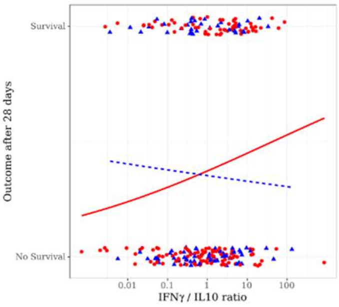

b)

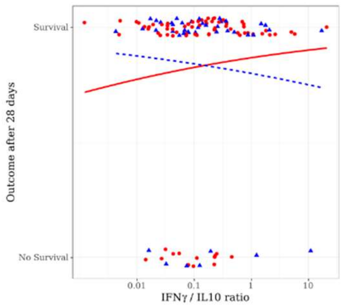

c)

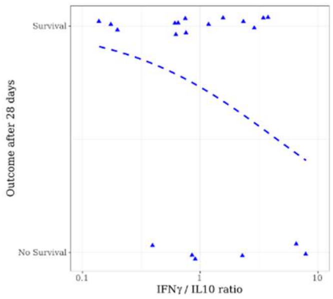

d)

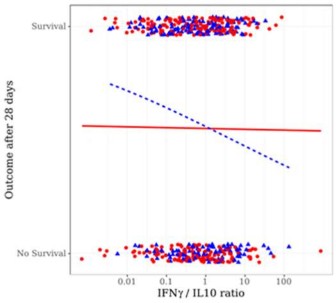

e)

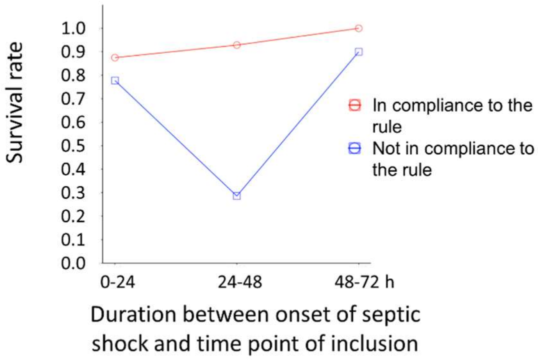

f)

Figure S2. Interaction plots of treatment and IFNγ/IL10 and of duration of septic shock

Interaction plot of treatment and IFNγ/IL10 in (a) the discovery set, CORTICUS, (b) in the validation set HSSG, (c) in the validation set SISPCT, (d) in the validation set Crossover study, and (e) using the pooled population. Each dot represents a patient (red: non HC-treated patients, blue triangles: HC-treated patients). The curves show the modeled distribution of survival according to the treatment (red line: non HC-treated, blue dashed line: HC-treated). Note that the overall survival rates are quite different between the cohorts. Details for the method are given in Text S9. f) Dependence of CORTICUS patient survival on the duration between the onset of septic shock and inclusion for the study for patients which were treated in compliance to the rule (red) and not in compliance to the rule (blue).

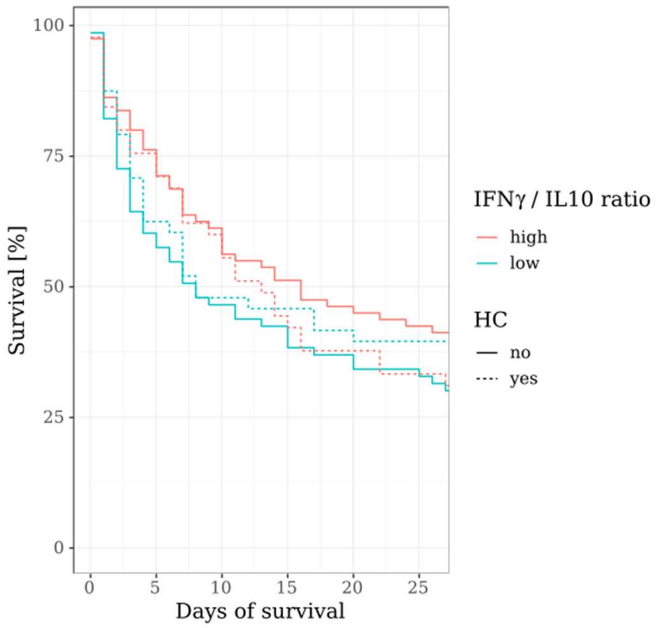

Figure S3. Change of survival rates of HSSG patients after HC treatment.

According to our rule, after day 10 HC treated patients with low ratio survived longer, whereas HC treated patients with high ratio showed a higher mortality.

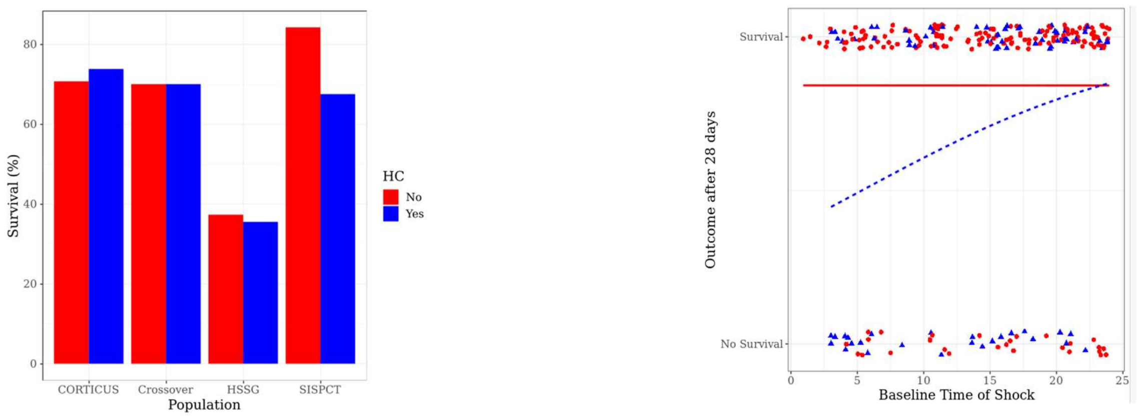

a) b)

Figure S4. Dependence of the survival rates of HC-treated SISPCT patients on the time interval between the beginning of shock and inclusion into the study

(a) 28-day-survival rates of HC-treated and not-HC-treated patients in the discovery set and in the validation sets. (b) Interaction plot of treatment and time between begin of septic shock and study inclusion for SISPCT patients. Each dot or triangle represents a patient (not HC treated patients: red dots; HC treated patients: blue triangles), whereas the curves show the modeled distribution of survival according to treatment (for not HC treated patients: red continuous line, for HC treated patients: blue dashed line).

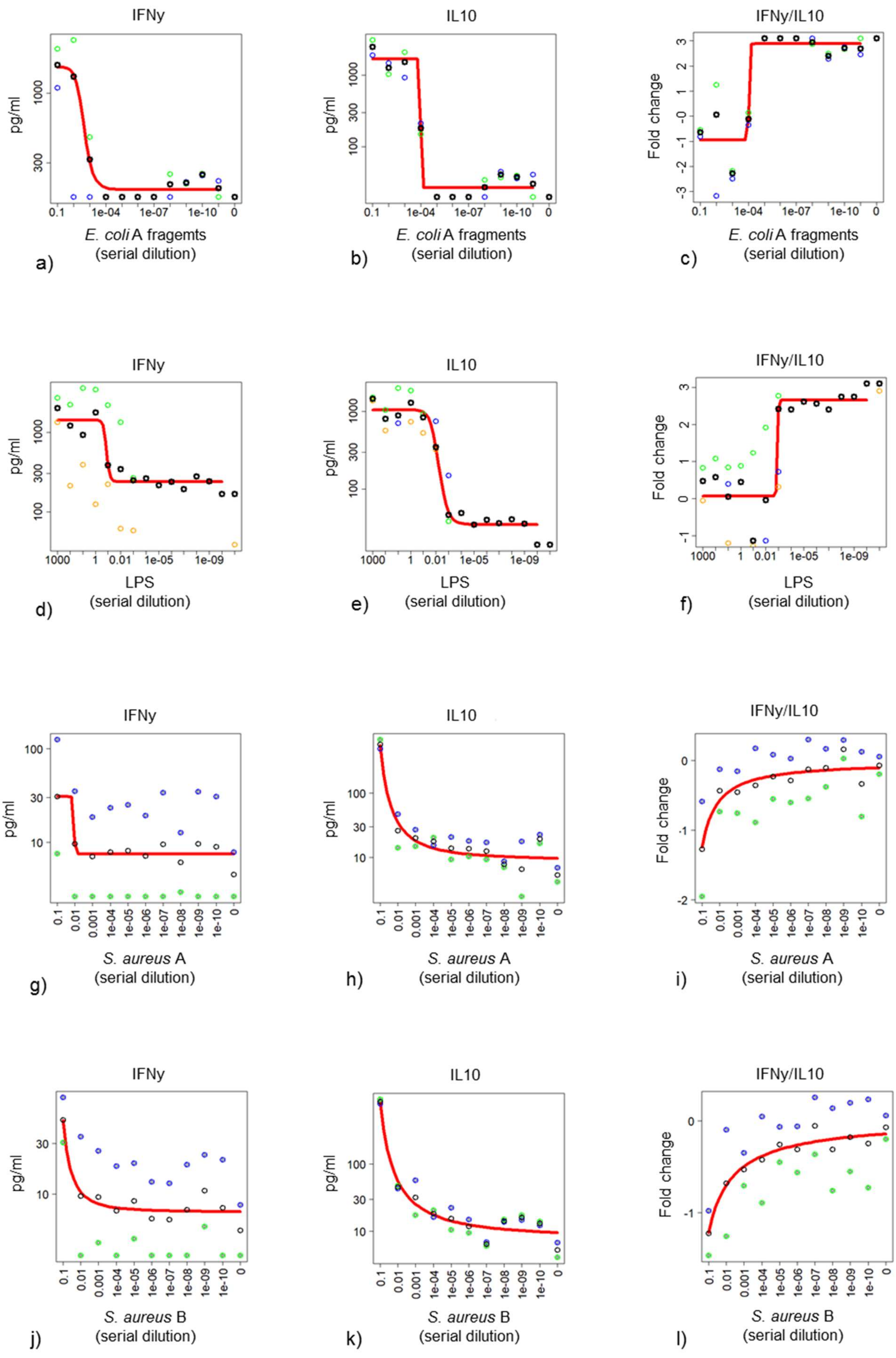
IFNy
IL10
IFNy/IL10
pg/ml
Fold change
E. coli A fragemts (serial dilution)
E. coli A fragments (serial dilution)
a)
b)
c)
LPS (serial dilution)
d)
e)
f)
S. aureus A (serial dilution)
g)
h)
i)
S. aureus B (serial dilution)
j)
k)
l)

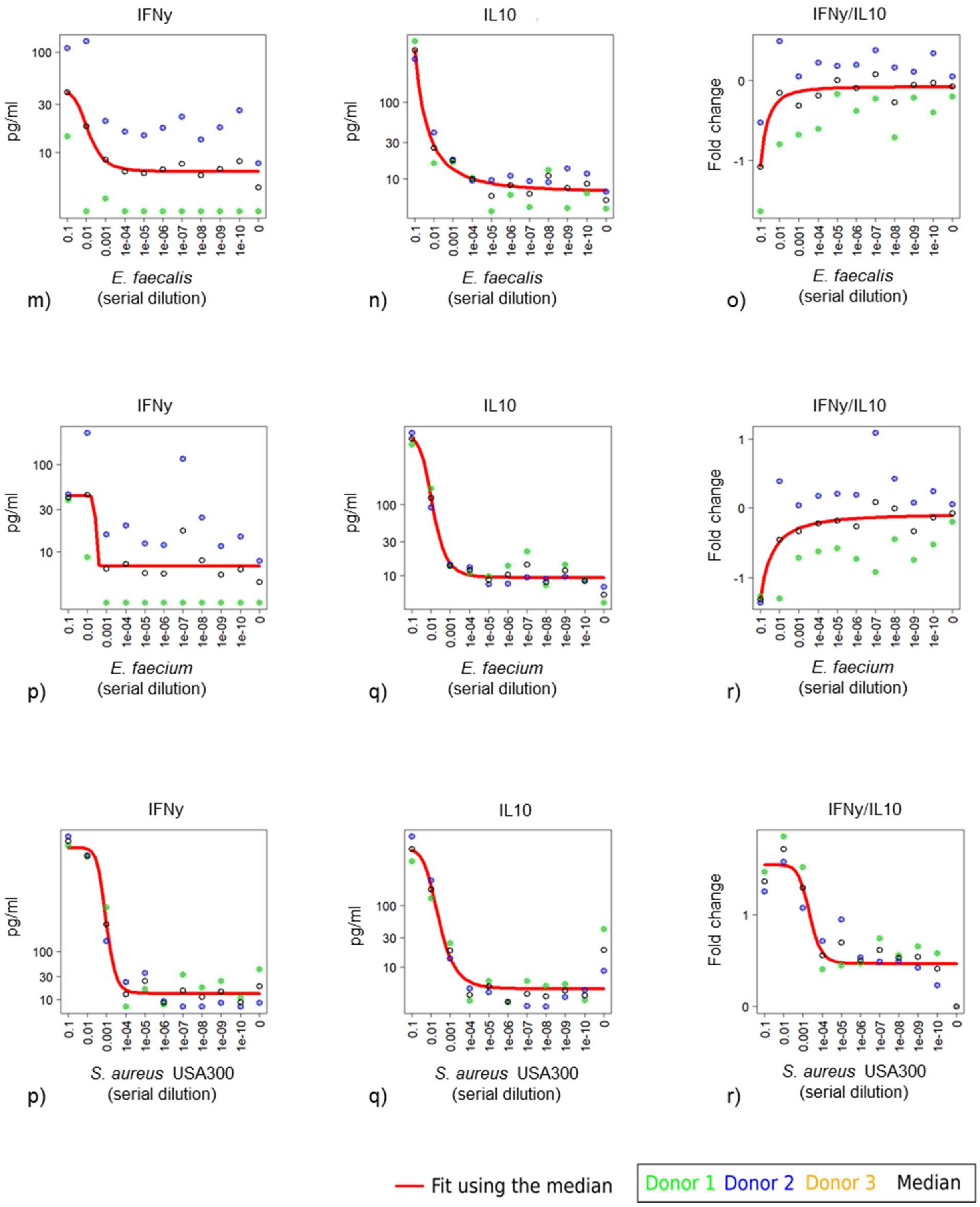
IFNy
IL10
IFNy/IL10
pg/ml
Fold change
E. faecalis
(serial dilution)
m)
n)
o)
E. faecium
(serial dilution)
p)
q)
r)
S. aureus USA300
(serial dilution)
p)
q)
r)
Fit using the median
Donor 1 Donor 2 Donor 3 Median

Figure S5. IFNγ, IL10 and IFNγ/IL10 of immune cells challenged *in vitro* with bacterial fragments or LPS

Whole blood from healthy donors (N = 2) was challenged with indicated serial dilution of sonicated and heat inactivated *E. coli* A fragments, a second clinical isolate, (a–c, results from the first clinical isolate are shown in the main text), LPS concentrations (d–f, 0.1 ng/ml to 1mg/ml), *S. aureus* A isolate (g-i), *S. aureus* B isolate (j-l), *E. faecalis* isolate (m-o), *E. faecium* isolate (p-r) or *S. aureus USA300* isolate (s-u) mimicking the immunologic loads. IFNγ and IL10 levels were measured 18h after immune induction in the supernatant. The responses of all clinical isolates and LPS are consistent with the results of the first clinical isolate displayed in the main text. Both IFNγ (a, d, g, j, m, p) and IL10 (b, e, h, k, n, q) were elevated with increasing challenge while IFNγ/IL10 (c, f, i, l, o, r) showed the opposite behaviour, i.e. a higher stimulus was associated with a lower ratio. The lab strain *S. aureus USA300* showed distinct response namely higher IFNγ levels (s) whereas IL10 (t) was similar, leading to high IFNγ/IL10 when the bacterial challenge was high.

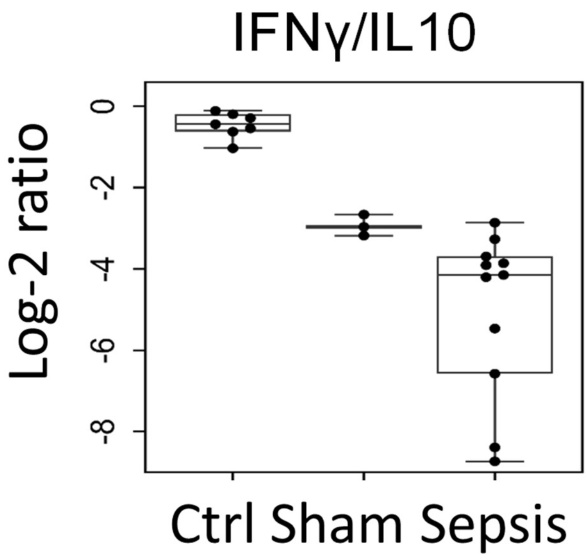

Figure S6. IFNγ/IL10 in septic mice and controls

Mice were challenged by cecal ligation and puncture (CLP) as well as peritoneal contamination and infection (PCI) and compared to untreated controls and sham treated mice. The ratio of IFNγ/IL10 was higher for the controls and lower for septic mice and was higher for the negative controls in comparison to the sham treated controls (t-tests: Untreated controls versus Sepsis: $p < 0.0001$; Sham versus Sepsis: $p = 0.0125$; Untreated controls versus Sham: $p = 0.0001$).

Supplementary Tables

Table S1. Detailed inclusion and exclusion criteria of CORTICUS, HSSG, SISPCT and the crossover study

Taken from references (3, 6, 8, 9)

Inclusion criteria:	
CORTICUS	1. Clinical evidence of infection within the previous 72 hours (may be present longer than 72 hours), only one of a, b, c, or d required:<br>a) Presence of polymorphonuclear cells in a normally sterile body fluid (excluding blood);<br>b) Positive culture or Gram staining of blood, sputum, urine or normal sterile body fluid;<br>c) Focus of infection identified by visual inspection (e.g. ruptured bowel with the presence of free air or bowel contents in the abdomen found at the time of surgery, wound with purulent drainage);<br>d) Other clinical evidence of infection-treated community acquired pneumonia, purpura fulminans, necrotising fascitis, etc.
	2. Evidence of a systemic response to infection as defined by the presence of two or more of the following signs within the previous 24 hours (these signs may be present longer than 72 hours):<br>a) Fever (temperature > 38.3°C) or hypothermia (rectal temperature < 35.6°C);<br>b) Tachycardia (heart rate of > 90 beat/min);<br>c) Tachypnea (respiratory rate > 20 breaths/min, $PaC0_2$ < 32 mmHg) or patient requires invasive mechanical ventilation;<br>d) Alteration of the WBC count: > 12,000 cells/mm^3, < 4,000 cells/mm^3 or > 10% immature neutrophils (bands).
	3. Evidence of shock defined by (a and b both required within the previous 72 hours (may NOT be present longer than 72 hours).<br>a) A systolic blood pressure < 90 mmHg or a decrease in SBP of more than 50 mmHg from baseline in previous hypertensive patients (for at least one hour) despite adequate fluid replacement OR need for vasopressors for at least one hour (infusion of dopamine ≥ 5 mg/kg/min or any dose of adrenaline, noradrenaline, phenylephrine or vasopressin) to maintain a SBP ≥ 90 mmHg;<br>b) Hypoperfusion or organ dysfunction which is not the result of underlying diseases or drugs, but is attributable to sepsis, including one of the following:<br>1. Sustained oliguria (urine output < 0.5 ml/kg/hr for a minimum of 1 hour)<br>2. Metabolic acidosis [pH of < 7.3, or a base deficit of ≥ 5.0 mmol/L, or an increased lactic acid concentration (> 2 mmol/L)].<br>3. Arterial hypoxemia ($Pa0_2/Fi0_2$ < 280 in the absence of pneumonia)($Pa0_2/Fi0_2$ < 200 in the presence of pneumonia).<br>4. Thrombocytopenia - platelet count ≤ 100,000 cells/mm^3.<br>5. Acute altered mental status (Glasgow Coma Scale < 14 or acute change from baseline).
	4. Age ≥ 18 years
	5. Informed Consent
	6. Measured cortisol level at baseline and 60 minutes after 0.25 mg cosyntropin stimulation
HSSG	1. Patients reclassified into infection and sepsis using the Sepsis-3 classification criteria (2,3)
	2. Age ≥ 18 years
	3. Informed Consent

	4. Community-acquired pneumonia and intraabdominal infections	
SISPCT	1. Onset of septic shock (C) less than 24 hours ago: Septic shock was defined as the presence of infection and SIRS as defined for severe sepsis as well as presence of arterial hypotension with a systolic blood pressure ≤ 90 mmHg or a mean arterial blood pressure ≤ 70 mmHg for at least 2 hours or administration of a vasopressor (dopamin ≥ 5 µg/kg/min; norepinephrine, epinephrine, phenylephrine, or vasopressin in any dosage) to maintain systolic blood pressure ≥ 90 mmHg or mean arterial blood pressure ≥ 70 mmHg despite adequate fluid loading.	
	3. Age ≥ 18 years	
	4. Informed consent	
Crossover	1. Presence of septic shock including,<br>a) Proven or strongly suspected infection<br>b) Three or more of these conditions: mechanical ventilation, heart rate of more than 90 beats per minute, temperature of more than 38°C or less than 36°C, a white blood cell count of more than 12,000 cells/µl or less than 4,000 cells/µl, or more than 10% immature cells<br>c) Sepsis-induced hypotension (systolic blood pressure of less than 90 mmHg or a reduction of more than 40 mmHg from baseline in the absence of other causes of hypotension)	
	2. Patients requiring norepinephrine to maintain a mean arterial pressure of more than 70 mmHg despite adequate fluid resuscitation.	
	2. Age ≥ 18 years	
	3. Informed Consent	
**Exclusion criteria:**		
CORTICUS	1. Pregnancy,<br>2. Age less than 18,<br>3. Underlying disease with a prognosis for survival of less than 3 months,<br>4. Cardiopulmonary resuscitation within 72 hours before study,<br>5. Drug-induced immunosuppression, including chemotherapy or radiation therapy within 4 weeks before the study,<br>6. Administration of chronic corticosteroids in the last 6 months or acute steroid therapy (any dose) within 4 weeks (including inhaled steroids). Topical steroids are not exclusions,<br>7. HIV positivity,<br>8. Presence of an advanced directive to withhold or withdraw life sustaining treatment (i.e. DNR),<br>9. Advanced cancer with a life expectancy less than 3 months,<br>10. Acute myocardial infarction or pulmonary embolus,<br>11. Another experimental drug study within the last 30 days,<br>12. Moribund patients likely to die within 24 hours,<br>13. Patients in the ICU for more than 2 months at the time of the start of septic shock,	
HSSG	1. Infection by the human immunodeficiency virus,<br>2. < 1,000 eutrophils/mm^3<br>3. Systemic intake of more than 0.3 mg/kg of equivalent prednisolone the last 15 days	
SISPCT	1. Pregnant or breast-feeding women,<br>2. Fertile female women without effective contraception,<br>3. Participation in interventional clinical trial within the last 30 days,<br>4. Current participation in any study,<br>5. Former participation in this trial,<br>6. Selenium intoxication,<br>7. No commitment to full patient support (i.e. DNR order),<br>8. Patient's death is considered imminent due to coexisting disease,<br>9. Relationship of the patient to study team member (i.e. colleague, relative),	

	10. Infection where guidelines recommend a longer duration of antimicrobial therapy (i.e. endocarditis, tuberculosis, malaria etc),<br>11. Immunocompromised patients,<br>12. Treatment with high-dose steroid in the last 5 days before begin of the study.<br>13. Onset of septic shock less than 3 hours before blood sampling for the cytokine measurements (see Methods)	
Crossover	1. Pregnancy,<br>2. Glucocorticoid medication within the last 3 months,<br>3. Ongoing immunosuppressive therapy,<br>4. Hematologic diseases,<br>5. Moribund state.	

Table S2. Duration between the onset of shock and the blood drawings in the CORTICUS sub-cohort

Patient	Time to HC (h)	Patient	Time to HC (h)	Patient	Time to HC (h)	Patient	Time to HC (h)
1	20	25	59	49	24	73	53.5
2	3	26	61	50	26	74	35
3	16	27	14.5	51	21	75	43
4	28.7	28	19	52	30	76	37.5
5	18	29	49	53	23	77	66.5
6	19	30	47	54	20	78	71
7	7	31	19	55	30	79	7
8	41.5	32	6	56	8.5	80	17.5
9	29	33	44	57	29	81	67
10	26	34	24	58	5	82	27.5
11	19	35	32	59	25	83	23
12	29	36	15	60	12	**Mean ± sd**	**29.4 ± 16.6**
13	58	37	16	61	23		
14	41	38	24	62	18		
15	13.5	39	39	63	39		
16	24.5	40	23	64	21		
17	5	41	21	65	24		
18	1.45	42	28	66	30		
19	45	43	24.5	67	30		
20	49	44	13	68	41		
21	56	45	42	69	53.5		
22	18	46	25.5	70	51		
23	67.5	47	26.5	71	51		
24	7	48	23	72	44		

Table S3. Patient characteristics of the analyzed HSSG cohort (n = 246)

HSSG	Non HC (n = 153)	HC (n = 93)
Gender (female)	63 (41%)	51 (55%)
Age, years (mean ± sd)	74 ± 13	72 ± 14
SOFA (mean ± sd)	9.5 ± 3.8	9.5 ± 3.7
APACHE II (mean ± sd)	24.5 ± 7.7	23.7 ± 7.5
28 days survival (n, %)	57 (37%)	33 (35%)
Site of infection (n, %)		
- Community-acquired pneumonia	95 (62%)	53 (57%)
- Intrabdominal infection	58 (38%)	40 (43%)
Co-morbidities (n, %)		
- History of diabetes mellitus type 2	39 (25%)	33 (35%)
- History of renal disease	19 (12%)	8 (9%)
- History of chronic heart failure	47 (31%)	24 (26%)
- History of chronic obstructive pulmonary disease	28 (18%)	15 (16%)

Table S4. Patient characteristics of the analysed SISPCT patients

a) All suitable patients from the placebo arm (n = 254)

Characteristics	Patients	
	HC-treated (n = 77)	Not treated (n = 177)
Sex (female, n, %)	28 (36%)	57 (32%)
Age, years (mean, 95% CI)	63 (59 66)	66 (64 68)
Weight, kg (mean, 95% CI)	82 (77 87)	82 (79 85)
Height, cm (mean, 95% CI)	172 (169 174)	172 (171 174)
On inotropes/pressors at day 0 (n, %)	76 (99%)	175 (99%)
Norepinephrine requirement, µg/kg/min at day 0 (mean, 95% CI)	0.73 (0.59 0.88)	0.29 (0.24 0.34)
Lactate, mmol/L at day 0 (mean, 95% CI)	5.8 (4.8 6.8)	2.8 (2.5 3.1)
SOFA (mean, 95% CI)	12.5 (11.7 13.2)	9.8 (9.4 10.2)
Day 28 (survivors, %)	52 (68%)	149 (84%)
Underlying infection (n, %)		
- Pneumonia	38 (49%)	69 (39%)
- Urogenital	9 (12%)	19 (11%)
- Abdominal	23 (30%)	61 (34%)
- Bone/Soft-tissue	3 (4%)	13 (7%)

b) Set of the balanced cohort

Characteristics	Patients	
	**HC-treated (n = 42)**	**Not treated (n = 76)**
Sex (female, n, %)	15 (36%)	22 (29%)
Age, years (mean, 95% CI)	62 (56 67)	67 (64 70)
Weight, kg (mean, 95% CI)	85 (77 93)	85 (80 89)
Height, cm (mean, 95% CI)	172 (169 176)	171 (169 173)
On inotropes/pressors at day 0 (n, %)	42 (100%)	74 (97%)
Norepinephrine requirement, µg/kg/min at day 0 (mean, 95% CI)	0.62 (0.45 - 0.78)	0.28 (0.22 0.33)
Lactate, mmol/L at day 0 (mean, 95% CI)	4.6 (3.7 5.5)	3.2 (2.8 3.7)
SOFA (mean, 95% CI)	12.2 (11.1 13.3)	10.6 (10.1 11.2)
Day 28 (survivors, %)	35 (83%)	64 (84%)
Underlying infection (n, %)		
- Pneumonia	21 (50%)	31 (41%)
- Urogenital	5 (12%)	8 (10%)
- Abdominal	17 (40%)	28 (37%)
- Bone/Soft-tissue	1 (2%)	2 (3%)

Table S5. List of all 137 predictors

42/14 Mean	IL10 (Con-A) [pg/ml]	Natural killer cells [%]	SOFA Resp
42/14 UR%	IL10 (LPS) [pg per 1000 cells]	Natural killer cells [1/nl]	T lymphocytes [%]
42/14ADP M	IL10 (LPS) [pg/ml]	Nitrit/Nitrat [µmol/l]	T lymphocytes [1/nl]
42/14ADP UR	IL10 [pg/ml]	Norepinephrine	T-Helper lymphocytes[%]
Age	IL10/TNFα ratio	OSF*_card	T-Helper lymphocytes[1/nl]
B lymphocytes [%]	IL10/TNFα ratio (LPS)	OSF_coag	T-Suppressor lymphocytes [%]
B lymphocytes [1/nl]	IL12/IFNγ ratio	OSF_liv	T-Suppressor lymphocytes [1/nl]
BE high	IL12/IL10 ratio	OSF_nerv	Temperature
BE low	IL12/TNFα ratio	OSF_renal	TH-Akt. Caspase 3 pos [%]
Bicarbonate high	IL6 (LPS) [pg per 1000 cells]	OSF_resp	TH-Akt. Caspase 3 pos [1/nl]
Bicarbonate low	IL6 (LPS) [pg/ml]	$PaCO_2$ high	TH-BCL-2 pos [% gated]
Bilirubin total high	IL6 [pg/ml]	$PaCO_2$ low	TH-BCL-2 pos [1/nl]
CD11b expression on PMN (Mean)	IL6/IFNγ ratio	PaO_2 low	Thrombocytes [1/nl]
Creatinine high	IL6/IFNγ ratio (LPS)	PEEP high	Tidal volume
D-Dimer [µg/ml]	IL6/IL10 ratio	pH high	Tidal volume [/kg]
DIC-Overall score	IL6/IL10 ratio (LPS)	pH low	TNFα (LPS) [pg per 1000 cells]
Dobutamine	IL6/IL12 ratio	Platelets high	TNFα (LPS) [pg/ml]
E-Selectin [pg/ml}	IL6/IL8 ratio	Platelets low	TNFR1
Factor VII [%]	IL6/TNFα ratio	PMNs [%]	TPZ [sec.]
FiO2 high	IL6/TNFα ratio (LPS)	PMNs [1/nl]	TS-Akt. Caspase 3 pos [%]
GCS	IL8 [pg/ml]	Protein C [%]	TS-Akt. Caspase 3 pos [1/nl]
Gender	IL8/IFNγ ratio	PTT high	TS-BCL2 pos [%]
HbO2 low	IL8/IL10 ratio	Respiratory rate	TS-BCL2 pos [1/nl]
Heart rate	IL8/IL12 ratio	SBP	Urea high
Hemoglobin high	IL8/TNFα ratio	Score D-Dim	Urinary output
HLA-DR expression on monocytes (Mean)	IL12 [pg/ml]	Score Thr	WBC high
HLA-DR-receptors on monocytes [1/cell]	INR	Score TPZ	WBC low
IFNγ (Con-A) [pg per 1000 cells]	Lactate high	Sedation	Weight
IFNγ (Con-A) [pg/ml]	Leukocytes [1/nl]	sFas	
IFNγ [pg/ml]	Lymphocytes [%]	SOFA	
IFNγ/IL10 ratio	Lymphocytes [1/nl]	SOFA Cardio	

*OSF: Organ system failure

Table S6. Pearson correlation of leukocyte counts to IFNγ, IL10 and IFNγ/IL10 ratio

	IFNγ		IL10		IFNγ/IL10 ratio	
**Cell type**	Correlation	P-value*	Correlation	P-value	Correlation	P-value
**Leukocytes [1/nl]**	-0.124	n.s.	-0.244	0.026	0.163	n.s.
**Lymphocytes [%]**	-0.091	n.s.	-0.026	n.s.	0.031	n.s.
**Lymphocytes [1/nl]**	-0.177	n.s.	-0.266	0.015	0.052	n.s.
**T lymphocytes [%]**	-0.137	n.s.	-0.230	0.036	-0.024	n.s.
**T lymphocytes [1/nl]**	-0.140	n.s.	-0.219	0.046	-0.036	n.s.
**T-Helper lymphocytes[%]**	-0.079	n.s.	-0.166	n.s.	-0.045	n.s.
**T-Helper lymphocytes[1/nl]**	-0.122	n.s.	-0.201	n.s.	-0.003	n.s.
**T-Suppressor lymphocytes [%]**	-0.129	n.s.	-0.187	n.s.	0.037	n.s.
**T-Suppressor lymphocytes [1/nl]**	-0.154	n.s.	-0.220	0.045	0.021	n.s.
**B lymphocytes [%]**	0.194	n.s.	0.240	0.029	-0.048	n.s.
**B lymphocytes [1/nl]**	-0.019	n.s.	-0.045	n.s.	0.031	n.s.
**Natural killer cells [%]**	-0.082	n.s.	0.013	n.s.	-0.041	n.s.
**Natural killer cells [1/nl]**	-0.226	0.040	-0.231	0.035	-0.057	n.s.
**Monocytes [%]**	-0.100	n.s.	0.046	n.s.	-0.088	n.s.
**Monocytes [1/nl]**	-0.169	n.s.	-0.235	0.033	0.010	n.s.

* P-values are not corrected for multiple testing.

Table S7. The lactate measurements available for the CORTICUS patients

Day 0	Day 1	Day 2	Day 3	Class
1.9	1.3	1.6	1	PL High-ratio
2.4	2.1	1.3	1.4	PL High-ratio
3.4	17	10	6	HC High-ratio
2.9	NA	NA	NA	PL Low-ratio
0.8	11	1.3	14	HC High-ratio
1.9	1.3	1	1.1	HC High-ratio
3.4	3.5	2.4	2.4	HC High-ratio
4.9	2.5	2.2	1.4	PL Low-ratio
7.2	2.1	1.4	1.8	PL High-ratio
4.1	3	1.2	1.4	HC High-ratio
0.9	0.3	0.2	0.1	PL High-ratio
1.4	NA	NA	NA	HC High-ratio
5.1	5	10.4	5.9	PL Low-ratio
2.3	2.5	1.6	1.4	HC Low-ratio
3.4	2.4	1.8	2.1	PL Low-ratio
5.5	1.4	1.6	1.3	PL Low-ratio
3.6	1.6	1.1	1.9	HC High-ratio

Day 0	Day 1	Day 2	Day 3	Class
2.5	NA	NA	NA	PL Low-ratio
0.6	NA	NA	NA	PL High-ratio
4	4	1.2	1.4	PL High-ratio
7.6	NA	NA	NA	HC Low-ratio
1.7	2.4	2.2	1.6	HC High-ratio
3	NA	NA	NA	PL High-ratio
1.2	NA	NA	NA	HC High-ratio
4.1	3.8	2.4	3.1	HC High-ratio
5.2	2.7	1.6	1.4	HC Low-ratio
1.8	2.5	3.1	5.4	HC High-ratio
1.6	1.5	1.4	1.6	PL High-ratio
6.3	5	2.2	0.5	HC High-ratio
1.1	1.3	1.4	1	PL High-ratio
1.5	NA	NA	NA	HC High-ratio
1.7	NA	NA	NA	PL Low-ratio
4.4	1.2	1.2	0.8	HC High-ratio
2.2	2.3	2.6	1.8	HC High-ratio
1	0.8	1.1	1.1	PL High-ratio
2.9	2.4	2.3	2.2	PL Low-ratio
2.7	2.4	2.2	2.1	HC Low-ratio
1.4	NA	NA	NA	PL High-ratio
1.7	NA	NA	NA	HC High-ratio
2	1.6	1.7	1	PL Low-ratio
1.4	1.9	2.2	3	PL Low-ratio
5	5	4.1	4.4	PL Low-ratio
3.6	2.4	1.5	1.5	PL Low-ratio
1	0.9	1	1	HC Low-ratio
1.6	1.2	1.1	0.9	PL High-ratio
1.1	17	9	12	PL Low-ratio
1.3	1.9	2.2	1.8	HC High-ratio
3.9	NA	NA	NA	PL Low-ratio
5.3	2.6	1.8	1.7	PL High-ratio
5.1	1.6	1.8	1.4	PL High-ratio
2.8	2.9	2.3	2	PL Low-ratio
3.7	2.7	2.1	2.9	PL Low-ratio
2.2	2.4	1.8	1.2	HC Low-ratio

Table S8. Patient characteristics of the analysed Crossover patients

**Characteristics**	**Early patients (n = 20)**
Age, years (mean, 95% CI)	54 (46, 63)
Sex (female, n, %)	7 (35%)
SAPS II (mean, 95% CI)	42 (35, 49)
SOFA (mean, 95% CI)	9.7 (8.5, 10.9)
28 day survival (n, %)	14 (70%)
Time between onset of septic shock and inclusion, hours (n, %)	
- < 24	4 (20%)
- 24 –48	7 (35%)
- 48 – 120	6 (30%)
- > 120	3 (15%)
Main source of infection (n, %)	
- Pulmonary	12 (60%)
- Gastrointestinal	8 (40%)
Microbiology	
- Gram positive	3 (15%)
- Gram negative	10 (50%)
- Mixed	3 (15%)
- Fungal	1 (5%)
- Not identified	3 (15%)

Table S9. Survival rates according to high and low IFNγ/IL10, restricted to patients with IFNγ or IL10 values within the detection limits

a) CORTICUS patients treated with *placebo*

	Non-survivors	Survivors	% Survivors
IFNγ/IL10 high	1	18	**95 %**☑*
IFNγ/IL10 low	9	8	**47 %**☒

b) CORTICUS patients treated with HC

	Non-survivors	Survivors	% Survivors
IFNγ/IL10 high	8	18	**69 %**☒
IFNγ/IL10 low	2	9	**82 %**☑

c) HSSG patients not treated with HC

	Non-survivors	Survivors	% Survivors
IFNγ/IL10 high	42	37	**47 %**☑
IFNγ/IL10 low	40	13	**25 %**☒

d) HSSG patients treated with HC

	Non-survivors	Survivors	% Survivors
IFNγ/IL10 high	30	12	**29 %**☒
IFNγ/IL10 low	24	15	**38 %**☑

e) SISPCT patients not treated with HC

	Non-survivors	Survivors	**% Survivors**
IFNγ/IL10 high	1	15	**94%**☑
IFNγ/IL10 low	12	32	**73%**☒

f) SISPCT patients treated with HC

	Non-survivors	Survivors	**% Survivors**
IFNγ/IL10 high	2	6	**75%**☒
IFNγ/IL10 low	5	21	**81%**☑

* ☑ Compliant to the rule; ☒ Non-compliant to the rule

References

1. Gurobi Optimization I. Gurobi Optimizer Reference Manual. 2016;at <http://www.gurobi.com>.
2. Singer M, Deutschman CS, Seymour CW, Shankar-Hari M, Annane D, Bauer M, Bellomo R, Bernard GR, Chiche J-D, Coopersmith CM, Hotchkiss RS, Levy MM, Marshall JC, Martin GS, Opal SM, Rubenfeld GD, van der Poll T, Vincent J-L, Angus DC. The Third International Consensus Definitions for Sepsis and Septic Shock (Sepsis-3). JAMA 2016;315:801.
3. Giamarellos-Bourboulis EJ, Tsaganos T, Tsangaris I, Lada M, Routsi C, Sinapidis D, Koupetori M, Bristianou M, Adamis G, Mandragos K, Dalekos GN, Kritselis I, Giannikopoulos G, Koutelidakis I, Pavlaki M, Antoniadou E, Vlachogiannis G, Koulouras V, Prekates A, Dimopoulos G, Koutsoukou A, Pnevmatikos I, Ioakeimidou A, Kotanidou A, Orfanos SE, Armaganidis A, Gogos C, Hellenic Sepsis Study Group. Validation of the new Sepsis-3 definitions: proposal for improvement in early risk identification. Clin Microbiol Infect 2017;23:104–109.
4. Benjamini Y, Hochberg Y. Controlling the False Discovery Rate: A Practical and Powerful Approach to Multiple Testing. J R Stat Soc 1995;57:289–300.
5. Ho D, Imai K, King G, Stuart E. MatchIt: Nonparametric Preprocessing for Parametric Causal Inference. J Stat Software, Artic 2011;42:1–28.
6. Keh D, Boehnke T, Weber-Cartens S, Schulz C, Ahlers O, Bercker S, Volk H-D, Doecke W-D, Falke KJ, Gerlach H. Immunologic and Hemodynamic Effects of “Low-Dose” Hydrocortisone in Septic Shock. Am J Respir Crit Care Med 2003;167:512–520.
7. Matera G, Puccio R, Giancotti A, Quirino A, Pulicari M, Zicca E, Caroleo S, Renzulli A, Liberto M, Focà A. Impact of interleukin-10, soluble CD25 and interferon-γ on the prognosis and early diagnosis of bacteremic systemic inflammatory response syndrome: a prospective observational study. Crit Care 2013;17:R64.
8. Sprung CL, Annane D, Keh D, Moreno R, Singer M, Freivogel K, Weiss YG, Benbenishty J, Kalenka A, Forst H, Laterre P-F, Reinhart K, Cuthbertson BH, Payen D, Briegel J.

Hydrocortisone Therapy for Patients with Septic Shock. N Engl J Med 2008;358:111–124.

9. Bloos F, Trips E, Nierhaus A, Briegel J, Heyland DK, Jaschinski U, Moerer O, Weyland A, Marx G, Gründling M, Kluge S, Kaufmann I, Ott K, Quintel M, Jelschen F, Meybohm P, Rademacher S, Meier-Hellmann A, Utzolino S, Kaisers UX, Putensen C, Elke G, Ragaller M, Gerlach H, Ludewig K, Kiehntopf M, Bogatsch H, Engel C, Brunkhorst FM, *et al.* Effect of Sodium Selenite Administration and Procalcitonin-Guided Therapy on Mortality in Patients With Severe Sepsis or Septic Shock. JAMA Intern Med 2016;176:1266.
